# Item-Weighted Likelihood Method for Measuring Growth in Longitudinal Study With Tests Composed of Both Dichotomous and Polytomous Items

**DOI:** 10.3389/fpsyg.2021.580015

**Published:** 2021-07-27

**Authors:** Xuemei Xue, Jing Lu, Jiwei Zhang

**Affiliations:** ^1^School of Mathematical Sciences, Xiamen University, Xiamen, China; ^2^Key Laboratory of Applied Statistics of MOE, School of Mathematics and Statistics, Northeast Normal University, Changchun, China; ^3^Key Lab of Statistical Modeling and Data Analysis of Yunnan Province, School of Mathematics and Statistics, Yunnan University, Kunming, China

**Keywords:** longitudinal model, item-weighted likelihood, mixed-format test, dichotomous item response, polytomous item response

## Abstract

In this paper, a new item-weighted scheme is proposed to assess examinees’ growth in longitudinal analysis. A multidimensional Rasch model for measuring learning and change (MRMLC) and its polytomous extension is used to fit the longitudinal item response data. In fact, the new item-weighted likelihood estimation method is not only suitable for complex longitudinal IRT models, but also it can be used to estimate the unidimensional IRT models. For example, the combination of the two-parameter logistic (2PL) model and the partial credit model (PCM, Masters, 1982) with a varying number of categories. Two simulation studies are carried out to further illustrate the advantages of the item-weighted likelihood estimation method compared to the traditional Maximum a Posteriori (MAP) estimation method, Maximum likelihood estimation method (MLE), Warm’s (1989) weighted likelihood estimation (WLE) method, and type-weighted maximum likelihood estimation (TWLE) method. Simulation results indicate that the improved item-weighted likelihood estimation method better recover examinees’ true ability level for both complex longitudinal IRT models and unidimensional IRT models compared to the existing likelihood estimation (MLE, WLE and TWLE) methods and MAP estimation method, with smaller bias, root-mean-square errors, and root-mean-square difference especially at the low-and high-ability levels.

## Introduction

The measurement of change has been a topic to both practitioners and methodologists (e.g., Dearborne, 1921; Woodrow, 1938; Lord, 1963; Fischer, 1973, 1976, 1995; Rasch, 1980; Andersen, 1985; Wilson, 1989; Embretson, 1991, 1997; von Davier and Xu, 2011; Barrett et al., 2015). Item response theory (IRT), particularly, a family of Rasch models (RM), provides a new perspective to modeling change. Andersen (1985) proposed the multidimensional Rasch model for modeling growth in the repeated administration of the same items at different occasions. Embretson (1991) presented a special multidimensional Rasch model for measuring learning and change (MRMLC) based on IRT. Embretson’s model postulated the involvement of *K* abilities for *K* occasions. Specifically, the MRMLC assumes that on the first occasion (*k* = 1), performance depends on initial ability. The MRMLC further assumes that on later occasions (*k* > 1), performance also depends on *k*−1 additional abilities, termed “modifiabilities,” as well as initial ability. Thus, the number of abilities increases at each time point. The same items are repeated over occasions in Andersen’s model which may lead to practice effects or memory effects and result in local dependency among item responses (von Davier and Xu, 2011), whereas items in Embretson’s MRMLC are not necessarily repeated. Fischer (2001) extended the MRMLC to polytomous items by extending the partial credit model (PCM, Masters, 1982). This paper extends Embretson’s method to measure growth based on item responses from mixed-format tests composed of both dichotomous and polytomous items which are frequently used in large-scale educational assessments, such as the National Assessment of Educational Progress (NAEP) and the Program for International Student Assessment (PISA). For polytomous items, each response category provides information. If categories within an item are close together, the item information will be peaked near the center of the location parameter of category. However, if the categories are spread further apart, each can add information at a different location. Therefore, the item information for a polytomous item can have multiple peaks and can be spread over a broader extent of the ability range. Thus, polytomous items may contain more information than dichotomous items (e.g., Donoghue, 1994; Embretson and Reise, 2000, p. 95; Jodoin, 2003; Penfield and Bergeron, 2005; Yao, 2009; Christine, 2010; Tao et al., 2012). How to utilize the potential information difference hidden in different item types to improve estimates of the latent trait is the main concern in our study.

As mentioned above, it has been demonstrated that polytomous items can often provide more information than dichotomous items concerning the level of estimated latent trait (Tao et al., 2012). Meanwhile, different items of the same type may provide different amount of information about latent trait estimation. To improve the precision of ability estimation, the aim of this study is to develop an efficient item-weighting scheme by assigning different weights to different items in accordance with the amount of information for a certain latent trait level. As early as 40 years ago, Lord (1980) has considered to optimal item weights for dichotomously scored items. Tao et al. (2012) proposed a bias-reduced item-weighted likelihood estimation method, and Sun et al. (2012) proposed weighted maximum-a-posteriori estimation, which focused on differentiating the information gained from different item types. In their methods, the weights were pre-assigned and known or automatically selected such that the weights assigned to the polytomous items are larger than that assigned to the dichotomous items. They assign different weights to different item types, instead of assigning different weights to different items, and items of the same type all have the same weight. For convenience, we called these weighting methods type-weighted estimation. However, different items of the same type may have different information for a certain latent trait level; the same weights assigned to the same-type items may not be statistically optimal in terms of the precision and accuracy of ability estimation due to neglecting the difference in the individual item contribution. It is expected that assigning a weight for each item based on its own contribution may increase measurement precision.

The remainder of this paper is organized as follows. First, we present the MRMLC and its polytomous extension, and then the proposed item-weighted likelihood estimation (IWLE) method and the other two ability estimation methods: Warm’s (1989) weighted likelihood estimation (WLE) and type-weighted maximum likelihood estimation (TWLE). Second, we show that the IWLE is consistent and asymptotically normal with mean zero and a variance-covariance matrix, and the bias of IWLE is of order *n*^−1^. Third, a simulation study is conducted to compare the proposed IWLE method with MLE, MAP, WLE, and TWLE. Fourth, a simulation study is conducted to show IWLE can also be applied to general unidimensional item response models. Finally, we conclude this paper with discussion.

## Materials and Methods

### MRMLC and Its Polytomous Extension

The MRMLC assumes that the probability of a correct response by person *l* on item *i* at occasion *k* can be written as:

(1) $$P\left(U_{ilk}=1|\left(\theta_{l1},\ldots ,\theta_{lk}\right),b_{i}\right)=\frac{\text{exp}\left(\sum_{v=1}^{k}\theta_{lv}-b_{i}\right)}{1+\text{exp}\left(\sum_{v=1}^{k}\theta_{lv}-b_{i}\right)},$$

where *U*_*ilk*_ is the response variable with values in {0,1}, *θ*_*l*1_ is the initial ability of person *l* on the first occasion *v* = 1, *θ*_*l*2_, …, *θ*_*lk*_ are modifiabilities that correspond to occasion *k* > 1, and *b_i_* is item difficulty Although the MRMLC may be applied to multiple occasions, for clarity, the model will be presented with only two occasions. To simplify the notation, the examinee subscript will not be shown in the following derivations. Using the abbreviated notations *P*_*i1*_ and *P*_*i2*_ for the probability of a correct item response for Occasions 1 and 2, respectively,

(2) $$P_{i1}\left(\theta_{1}\right)=\frac{\text{exp}\left(\theta_{1}-b_{i}\right)}{1+\text{exp}\left(\theta_{1}-b_{i}\right)},$$

and

(3) $$P_{i2}\left(\theta_{1},\theta_{2}\right)=\frac{\text{exp}\left(\theta_{1}+\theta_{2}-b_{i}\right)}{1+\text{exp}\left(\theta_{1}+\theta_{2}-b_{i}\right)},$$

Regarding the polytomous items, we use the abbreviated notations *P*_*ij*1_ and *P*_*ij*2_ to denote the probability of selecting response category *j* (where *j* = 1, …, *h*) of polytomous item *i* for Occasions 1 and 2, respectively,

(4) $$P_{ij1}\left(\theta_{1}\right)=\frac{\text{exp}\left(j\theta_{1}-\sum_{v=1}^{j}b_{iv}\right)}{\sum_{r=1}^{h}\text{exp}\left(r\theta_{1}-\sum_{v=1}^{r}b_{iv}\right)},$$

and

(5) $$P_{ij2}\left(\theta_{1},\theta_{2}\right)=\frac{\text{exp}\left[j\left(\theta_{1}+\theta_{2}\right)-\sum_{v=1}^{j}b_{iv}\right]}{\sum_{r=1}^{h}\text{exp}\left[r\left(\theta_{1}+\theta_{2}\right)-\sum_{v=1}^{r}b_{iv}\right]},$$

To develop a conditional maximum likelihood estimation method for item parameters in the learning process model, Embretson (1991) constructed a data design structure for item calibration in which item blocks are counterbalanced in several occasions over groups. This data design matrix is needed to determine the occasion on which an item appears for an individual. Every item must be observed on every occasion, but to preserve local independence, an item should be administered only once to an individual across the two occasions. To incorporate Embretson’s design structure, two groups of examinees are asked to respond to unique items on two occasions, *k*_*ig*_ is now defined as a binary variable to indicate the occasion on which item *i* is administered to group *g*(*g* = 1,2).

Specifically,

kig={1,ifitemiisadministeredingroupgunderOccasion 1,0,ifitemiisadministeredingroupgunderOccasion 2.

Thus, the probability of a response vector **u** = (*u*_1_, …, *u*_*n*_) in group *g*, *P*_*g*_ for *n* items conditional on ability vector (*θ*_1_, *θ*_2_), item difficulty vector **b** and item occasion vector *k_g_*, for *k*_1*g*_, …, *k*_*ng*_ is given by:

$$\begin{array}{c} P_{g}\left(\text{U}=\text{u}|\left(\theta_{1},\theta_{2}\right),\text{b},k_{g}\right)\\ \;=\prod_{i=1}^{n}{\left[P_{i1}{\left(\theta_{1}\right)}^{u_{i}}{\left(1-P_{i1}\left(\theta_{1}\right)\right)}^{1-u_{i}}\right]}^{k_{ig}}\cdot \\ \;{\left[P_{i2}{\left(\theta_{1},\theta_{2}\right)}^{u_{i}}{\left(1-P_{i2}\left(\theta_{1},\theta_{2}\right)\right)}^{1-u_{i}}\right]}^{1-k_{ig}}, \end{array}$$

where **b** = (*b*_1_, …, *b*_*n*_).

First, suppose that person *l* is assigned to a test condition group *g* that receives items **I**. For the following considerations, it is assumed that some of the items **I** = {*I*_1_, …, *I*_*n*_} are presented at time point (Occasion) 1, called the “pretest,” denoted **I**_1_, and some items are presented at point time 2, called the “posttest,” denoted **I**_2_ according to Fischer (2001). The nonempty item subsets **I**_1_ and **I**_2_ may be completely different, may overlap, or may be identical. For convenience, however, a notation is adopted where **I**_1_ and **I**_2_ are considered disjoint subsets of **I**,**I**_1_ = {*I*_1_, …, *I*_*n*_1__} and **I**_2_ = {*I*_*n*_1_ + 1_, …, *I*_*n*_}. However, the cases in which **I**_1_ and **I**_2_ overlap are implicitly covered; it suffices to let some *I*_*a*_ ∈ **I**_1_ have the same parameters as some *I*_*b*_ ∈ **I**_2_. Let us consider mixed-format tests; specifically, *k* items *I*_1_, …, *I*_*k*_ are dichotomous and *n*_1_−*k* items *I*_*k* + 1_, …, *I*_*n*_1__ are polytomous in the pretest; for the posttest, *m*−*n*_1_ items *I*_*n*_1_ + 1_, …, *I*_*m*_ are dichotomous and *n*−*m* items *I*_*m* + 1_, …, *I*_*n*_ are polytomous.

### Maximum Likelihood Estimator

Now we consider the problem of likelihood estimation of ability θ = (*θ*_1_, *θ*_2_). The likelihood function of responses is the product of two types of likelihood functions given local independence:

(6) $$L\left(\theta |\text{U}\right)=L_{d}\left(\theta |\text{U}\right)L_{p}\left(\theta |\text{U}\right),$$

where

(7) $$\begin{array}{c} L_{d}\left(\theta |\text{U}\right)=\left(\prod_{i=1}^{k}P_{i1}{\left(\theta_{1}\right)}^{u_{i}}Q_{i1}{\left(\theta_{1}\right)}^{1-u_{i}}\right)\cdot \\ \;\left(\prod_{i=n_{1}+1}^{m}P_{i2}{\left(\theta_{1},\theta_{2}\right)}^{v_{i}}Q_{i2}{\left(\theta_{1},\theta_{2}\right)}^{1-v_{i}}\right), \end{array}$$

and

(8) $$L_{p}\left(\theta |\text{U}\right)=\left(\prod_{i=k+1}^{n_{1}}\prod_{j=1}^{h}P_{ij1}{\left(\theta_{1}\right)}^{u_{ij}}\right)\cdot \left(\prod_{i=m+1}^{n}\prod_{j=1}^{h}P_{ij2}{\left(\theta_{1},\theta_{2}\right)}^{v_{ij}}\right),$$

are the likelihood functions of the dichotomous model and the polytomous model of a mixed-format longitudinal test, respectively, in which,

$$Q_{i1}\left(\theta_{1}\right)=1-P_{i1}\left(\theta_{1}\right),Q_{i2}\left(\theta_{1},\theta_{2}\right)=1-P_{i2}\left(\theta_{1},\theta_{2}\right).$$

The response matrix **U** contains the responses to dichotomous items *u*_*i*_, *v*_*i*_ and the responses to polytomous items *u*_*ij*_, *v*_*ij*_. The conventional maximum likelihood estimator (MLE) $\hat{\theta }$ can be obtained by maximizing the log-likelihood function log*L*(**θ**|*U*).

### Weighted Likelihood Estimator

Warm (1989) proposed a weighted likelihood estimation (WLE) method for dichotomous IRT model. Compared with the maximum likelihood estimation, Warm’s weighted likelihood estimation method can obtain less bias estimation. Penfield and Bergeron (2005) extended this method to the case of the generalized partial credit model (GPCM). The weighted likelihood function of a mixed-type model can be expressed as:

$$w\left(\theta \right)L\left(\theta |\text{U}\right)=w\left(\theta \right)L_{d}\left(\theta |\text{U}\right)L_{p}\left(\theta |\text{U}\right),$$

where *w*(θ) is the weighting function, $w\left(\theta \right)=I^{\frac{1}{2}}$ in one or two parameter models of IRT. *w*(θ) is multiplied by the likelihood function *L*(**θ**|**U**), and the product is maximized. WLE was proved to yield asymptotically normally distributed estimates, with finite variance, and with bias of only *o*(*n*^−1^).

### Item-Weighted Maximum Likelihood Estimator

In this section, we consider the following item-weighted likelihood function:

(9) $$IWL\left(\theta |\text{U}\right)=IWL_{d}\left(\theta |\text{U}\right)\cdot IWL_{p}\left(\theta |\text{U}\right),$$

where

(10) $$\begin{array}{c} IWL_{d}\left(\theta |U\right)=\prod_{i=1}^{k}{\left\{P_{i1}{\left(\theta_{1}\right)}^{u_{i}}\cdot Q_{i1}{\left(\theta_{1}\right)}^{1-u_{i}}\right\}}^{w_{i}\left(\theta \right)}\cdot \\ \;\prod_{i=n_{1}+1}^{m}{\left\{P_{i2}{\left(\theta_{1},\theta_{2}\right)}^{v_{i}}\cdot Q_{i2}{\left(\theta_{1},\theta_{2}\right)}^{1-v_{i}}\right\}}^{w_{i}\left(\theta \right)}, \end{array}$$

and

(11) $$\begin{array}{c} IWL_{p}\left(\theta |\text{U}\right)=\prod_{i=k+1}^{n_{1}}{\left\{\prod_{j=1}^{h}P_{ij1}{\left(\theta_{1}\right)}^{u_{ij}}\right\}}^{w_{i}\left(\theta \right)}\cdot \\ \;\prod_{i=m+1}^{n}{\left\{\prod_{j=1}^{h}P_{ij2}{\left(\theta_{1},\theta_{2}\right)}^{v_{ij}}\right\}}^{w_{i}\left(\theta \right)}, \end{array}$$

are the item-weighted likelihood functions of the dichotomous model and the polytomous model of a mixed-format longitudinal test, respectively. Here the weight vector:

(*w*_1_(**θ**), …, *w*_*n*_(**θ**)) satisfy *w*_*i*_(**θ**) > 0 for each *i* and $\sum_{i=1}^{n}w_{i}\left(\theta \right)=1$.

Note that,

(12) $$w_{i}\left(\theta \right)=\frac{I_{i}\left(\theta \right)}{I\left(\theta \right)},\mathrm{for}\mathrm{all}i\in \left\{1,2,\ldots ,n\right\},$$

where *I*_*i*_(**θ**) is the information function of item *i* given as:

$$I_{i}\left(\theta \right)=\left\{ \begin{array}{cc} P_{i}\left(\theta \right)Q_{i}\left(\theta \right), & \\ & \mathrm{for dichotomous item}i,\\ \sum_{j=1}^{h}j^{2}P_{ij}\left(\theta \right)-{\left(\sum_{j=1}^{h}jP_{ij}\left(\theta \right)\right)}^{2}, & \\ & \mathrm{for polytomous item}i. \end{array}\right.$$

*P_i_* is the probability of a correct response to item *i*, *Q*_*i*_ = 1−*P*_*i*_, *P*_ij_ is the probability of selecting response category *j* (where *j* = 1, …, *h*) of polytomous item *i*, and $I\left(\theta \right)=\sum_{i=1}^{n}I_{i}\left(\theta \right)$ is the test information function consisting both dichotomous and polytomous items (Muraki, 1993). Using the information ratio of each item to the test at a certain ability level, the weights of items are determined.

In IRT, the item and test information functions relate to how well an examinee’s ability is being estimated over the whole ability scale; they are usually used to calculate the standard error of measurement and the reliability. Since the test information is a function of proficiency (or whatever trait or skill is measured) and the items on the test, the expression of the proposed weights involves the ability level **θ** and item characteristic parameters. The weights may be “adaptive” in the sense that they are allowed to be estimated based on the ability level and individual test items. By using the information ratio of each item to the test to determine the weights, so the more information an item has at a certain ability level, the larger weight could be assigned to it. According to the proposed weighting method, the weight for the polytomous item is then larger than that for the dichotomous item and the weights for the same type item are different due to the difference between the amounts of item information. The weight assigned to each item just indicates its contribution to the precision for ability parameter estimation. This item weighting scheme maximizes the information obtained from both different types of items and different items of the same type and may lead to more accurate estimates of the latent trait than equally weighting all items. If each item with same scoring procedure has same item information at a certain latent trait level, the weights are equal between them. Hence, the proposed item-weighted likelihood method may be an extension of the method proposed by Tao et al. (2012). The item-weighted likelihood estimator (IWLE) can be obtained by maximizing the item-weighted log-likelihood function log*IWL*(**θ**|**U**) (for derivation details, see Supplementary Appendix A). Maximum likelihood estimator (Lord, 1983) was shown to have bias of *O*(*n*^−1^). When the weights are determined at a certain ability level, with some assumptions made by Lord (1983), the bias of the item-weighted maximum likelihood estimation also has bias of *O*(*n*^−1^). The approach and techniques of this derivation were taken from, and parallel closely, the derivations in Lord (1983). The asymptotic properties of IWLM can be obtained by generalizing those of Bradley and Gart (1962) (for more details, see Supplementary Appendix B).

### Type-Weighted Maximum Likelihood Estimator

In contrast to the MLE, the type-weighted maximum likelihood estimator (TWLE) yields usable ability estimator for mixed-type tests composed of both dichotomous and polytomous items (Sun et al., 2012). The type-weighted likelihood function of a mixed-type model can be expressed as:

$$TWL\left(\theta |\text{U}\right)=L_{d}{\left(\theta |\text{U}\right)}^{{\tilde{w}}_{1}\left(\theta \right)}L_{p}{\left(\theta |\text{U}\right)}^{{\tilde{w}}_{2}\left(\theta \right)},$$

where

$${\tilde{w}}_{1}\left(\theta \right)={\left(\frac{I_{d}\left(\theta \right)}{I\left(\theta \right)}\right)}^{\alpha },{\tilde{w}}_{2}\left(\theta \right)={\left(\frac{I_{p}\left(\theta \right)}{I\left(\theta \right)}\right)}^{\beta },$$

$$I=I_{d}+I_{p},$$

$I_{d}=\sum_{i=1}^{k}I_{i}+\sum_{i=n_{1}+1}^{m}I_{i}$, and $I_{p}=\sum_{i=k+1}^{n_{1}}I_{i}+\sum_{i=m+1}^{n}I_{i}$, are test information of the dichotomous and polytomous model based on the longitudinal model, respectively. According to the weighting scheme proposed by Sun et al. (2012), the ratio parameters α, β determined to make sure that the weight assigned to the polytomously scored item is larger than that assigned to the dichotomously scored item. Three steps are needed to determine the ratio parameters α, β and the two weights. First, we obtain the ML estimator ${\hat{\theta }}_{0}$ and take it as the initial estimator. Second, if $I_{d}\left({\hat{\theta }}_{0}\right)<I_{p}\left({\hat{\theta }}_{0}\right)$, the two ratio parameters are all equal to 1. Otherwise, we may set α and β to be a small value ε (such as ε < 0.4) to make sure $I_{d}\left({\hat{\theta }}_{0}\right)<I_{p}\left({\hat{\theta }}_{0}\right)$. Then, no change is needed for either α or β if ${\tilde{w}}_{1}\left({\hat{\theta }}_{0}\right)<{\tilde{w}}_{2}\left({\hat{\theta }}_{0}\right)$. Otherwise, we may increase α in increments of 0.05 or less, or decrease β in increments of 0.05 or less. We adjust α and β to ensure ${\tilde{w}}_{1}\left({\hat{\theta }}_{0}\right)<{\tilde{w}}_{2}\left({\hat{\theta }}_{0}\right)$. Third, we maximize the type-weighted log-likelihood function log*TWL*(**θ**|**U**) to obtain $\hat{\theta }$ with the obtained α and β values from the above. If ${\tilde{w}}_{1}\left(\hat{\theta }\right)<{\tilde{w}}_{2}\left(\hat{\theta }\right)$, the $\hat{\theta }$ is the TWLE. Otherwise, the ratio parameters should be adjusted continually basing on the above process until ${\tilde{w}}_{1}\left(\hat{\theta }\right)<{\tilde{w}}_{2}\left(\hat{\theta }\right)$.

The above three-weighted estimations TWLE, WLE, and IWLE have different weighting schemes. For TWLE, the larger weights are assigned to the polytomous items and the smaller weights are assigned to the dichotomous items. This method only assigns different weights to different item types, instead of assigning different weights to different items, thus items of the same type all have the same weight. However, different items of the same type may have different information about a certain latent trait level; the same weights assigned to the same-type items may not be statistically optimal in terms of the precision and accuracy of ability estimation due to neglecting the difference in the individual item contribution. The proposed IWLE assigns different weights to different items in accordance with the amount of the information an item provides at a certain latent trait level. Using the information ratio of each item to the test, the weights of items are determined. This improved IWLE procedure that incorporates item weights in likelihood functions for the ability parameter estimation may increase measurement precision. The WLE provides a bias correction to the maximum likelihood method. The weight function is multiplied by the likelihood function *L*(**θ**|**U**) in the WLE method, which provides a correction to the maximum likelihood estimation method by solving an weighted, log-likelihood equation. The WLE and IWLE are both consistent and asymptotically normal with mean zero and a variance-covariance matrix, and the bias of the estimators is of order *n*^−1^.

## Simulation Study 1

### Simulation Design

In this section, the performance of the three weighting methods, the WLE, the type-weighted likelihood estimation (TWLE), and IWLE are compared. To investigate the effects of the test-length and the proportion of dichotomous and polytomous items in a mixed-format test on the properties of the **θ** estimators, nine artificial tests were constructed at each time point, three of them short (10 items with 7, 5, and 3 dichotomous items), three medium (30 items with 20, 15, and 10 dichotomous items), and three long (60 items with 40, 30, and 20 dichotomous items). In the simulation, the 3 levels of test length were representative of those encountered in measuring settings using fixed-length tests. The 3 levels of proportion of dichotomous and polytomous items (λ = 2,1,0.5) were selected, so that we may have a thorough investigation into the property of different weighting methods.

The item parameters and ability parameters are set as follows. The difficulty parameters of the dichotomous items were randomly generated from the standard normal distribution *N*(0,1). The polytomously scored items with four-category were constructed. The step parameters of each polytomous item were randomly generated from four normal distributions:

b_*i*1_ ∼ *N*(−1.5, 0.2), *b*_*i*2_ ∼ *N*(−0.5, 0.2), *b*_*i*3_ ∼ *N*(0.5, 0.2), and *b*_*i*4_∼*N*(1.5, 0.2).

This pattern of location parameters centers items on zero and thus centers the test on zero. In the simulation, 17 equally spaced *θ*_1_ values were considered, ranging from –4.0 to 4.0 in increments of 0.5. We set 3 values of *θ*_2_(0.6,0.8, and 1.0) for 3 different initial ability levels: high (value of *θ*_1_ larger than 2), medium (value of *θ*_1_ between –2 and 2), and low (value of *θ*_1_ smaller than –2), respectively. Thus, a high initial ability will have low gain, a medium initial ability will have moderate gain, and a low initial ability will have high gain. At each level of (*θ*_1_, *θ*_2_), *N*(*N* = 1000) replications were administered for all 9 tests. In each replication, the dichotomous item responses were simulated according to the MRMLC model as presented in Equations 2 and 3, and the polytomous item responses were simulated according to the PCM as presented in Equations 4 and 5. For the tests containing response patterns consisting of all correct responses for dichotomous items and all 4s for polytomous items or all incorrect responses for dichotomous items and all 4s, the Newton-Raphson algorithm cannot converge, and thus the likelihood estimators could not be obtained. These response patterns were removed from the analysis, and the same item responses were scored using the WLE, TWLE, and IWLE procedures. In the simulation, the **θ** in the weight for each item is taken as $\hat{\theta }$, the MLE of **θ**. All levels of the number of items, the proportion of dichotomous and polytomous items, and the number of examinee were crossed, resulting in 27 conditions of test properties at each time point. For each of the 27 conditions of test properties, the WLE, TWLE, and IWLE were obtained for each of the response patterns.

### Evaluation Criteria

The bias, absolute bias, root mean squared error (RMSE) and root mean squared difference (RMSD) of the ability estimates were used as evaluation criteria to examine all estimation methods. The absolute bias is calculated using Equation 13. In Equation 13,θ denotes the true ability value and ${\hat{\theta }}_{l}$ the corresponding ability estimate for the *l* th replication.

(13) $$\left|Bias\right|=\left|\frac{1}{N}\sum_{i=1}^{N}\left({\hat{\theta }}_{l}-\theta \right)\right|$$

RMSE and RMSD are calculated using Equation 14 and 15, respectively:

(14) $$RMSE=\sqrt{\frac{1}{N}\sum_{l=1}^{N}{\left({\hat{\theta }}_{l}-\theta \right)}^{2}},$$

(15) $$RMSD=\sqrt{\frac{1}{N}\sum_{l=1}^{N}{\left({\hat{\theta }}_{l}-\frac{1}{N}\sum_{l=1}^{N}{\hat{\theta }}_{l}\right)}^{2}}.$$

*N* is the number of replications. In simulation studies, we fix the number of replications at 1000, that is, *N* = 1000.

### Results of Simulation

**FIGURE 1:**
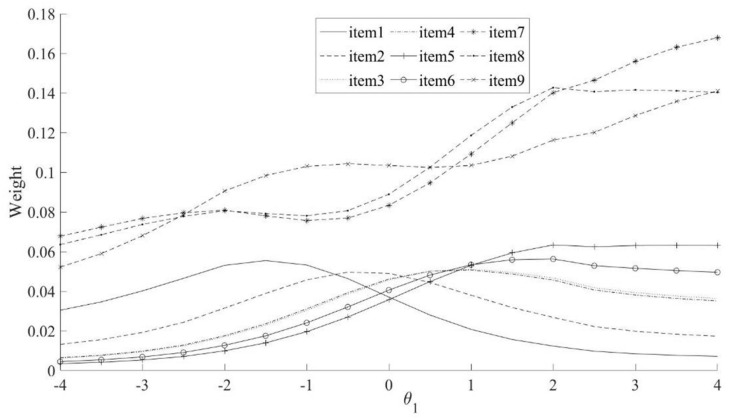
The weights of IWLE based on *θ*_1_ for dichotomous items (item 1 to 6) and polytomous items (items 7 to 9) in test 1.

**FIGURE 2:**
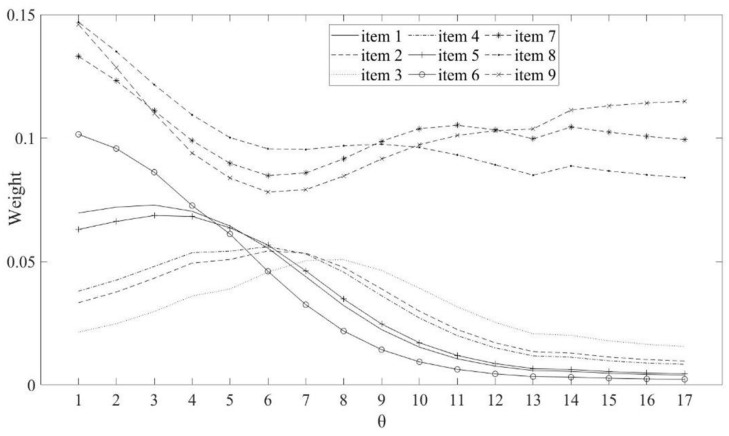
Weights based on θ(θ = (*θ*_1_, *θ*_2_)) at 17 ability levels for dichotomous items (item 1 to 6) and polytomous items (items 7 to 9) in test 2.

The weights of IWLE for 6 dichotomous and 3 polytomous items are shown in Figures 1, 2 The purpose of these figures is to give more intuition in terms of our item weighting scheme. The weights are based on the individual test items and the ability level, with *θ*_1_ ranging from –4.0 to 4.0 and 3 values of *θ*_2_(0.6,0.8, and 1.0). We can find that the different items are designed with different weights. In addition, the weights assigned to polytomous items are larger than that of dichotomous items.

Table 1 shows the correlation between the estimated abilities and the true abilities for all three weighting estimation methods under nine conditions. The higher degree of correlation obtained by the IWLE ability estimates indicates that the IWLE produces better quality ability estimates. The results in Table 1 indicate that the longer tests provide higher correlation between the estimated abilities and the true abilities. In the tests with the same length, higher proportion of polytomous and dichotomous items also provide higher correlation between the estimated abilities and the true abilities.

**TABLE 1:** Correlation between the estimated abilities and the true abilities for all three weighting estimation methods under nine conditions.

		Test								
N	Method	7d+3p	5d+5p	3p+7d	20d+10p	15d+15p	10d+20p	40d+20p	30d+30p	20d+40p
1000	IWLE	0.8685	0.9068	0.9189	0.9478	0.9548	0.9593	0.9663	0.9609	0.9822
	WLE	0.8189	0.8378	0.8608	0.9246	0.9375	0.9470	0.9640	0.9606	0.9716
	TWLE	0.8001	0.8344	0.8542	0.9216	0.9360	0.9451	0.9612	0.9796	0.9711

*n_1_d + n_2_p means the (n_1_ + n_2_) -item test with n_1_ dichotomous items and n_2_ polytomous items.*

The simulation results of 3 test lengths show similar trends for the three weighting estimators: WLE, TWLE, and IWLE. Due to page limitation, only those for the 30-item test are presented. The complete results can be obtained from the author. Tables 2–7 displays the obtained values of absolute bias, and RMSD for WLE, TWLE, and IWLE at 17 different levels of initial ability *θ*_1_(−4,−3.5,,3.5,4) and 3 different levels of growth *θ*_2_(0.6,0.8,1) in the simulation scenarios.

**TABLE 2:** Absolute bias and root mean squared difference for WLE, TWLE, and IWLE at 17 different levels of initial ability on 20d+10p.

20d+10p	Methods					
*N* = 1000	IWLE		WLE		TWLE	
θ_*1*_	Abs.Bias	RMSD	Abs.Bias	RMSD	Abs.Bias	RMSD
−4.0	0.5615	1.7379	1.1045	3.5101	1.1596	3.5370
−3.5	0.3221	1.3011	0.4678	2.3189	0.5249	2.3495
−3.0	0.1402	0.9134	0.1582	1.3797	0.1783	1.4074
−2.5	0.0342	0.5038	0.0361	0.5118	0.0494	0.5675
−2.0	0.0162	0.4809	0.0130	0.4401	0.0158	0.4931
−1.5	0.0047	0.4384	0.0005	0.4061	0.0054	0.4494
−1.0	0.0045	0.4020	0.0004	0.3821	0.0049	0.4237
−0.5	0.0047	0.3662	0.0119	0.3570	0.0053	0.3943
0.0	0.0092	0.3718	0.0114	0.3433	0.0107	0.3784
0.5	0.0071	0.3707	0.0041	0.3456	0.0083	0.3740
1.0	0.0076	0.3654	0.0039	0.3378	0.0078	0.3670
1.5	0.0179	0.3834	0.0164	0.3675	0.0189	0.4095
2.0	0.0112	0.4025	0.0039	0.3764	0.0169	0.4272
2.5	0.0205	0.4133	0.0187	0.4400	0.0455	0.4926
3.0	0.0190	0.5846	0.0282	0.6321	0.0508	0.6763
3.5	0.2811	1.1297	0.3414	2.0295	0.3876	2.0387
4.0	0.3805	1.3812	0.6268	2.8406	0.6900	2.8470

*20d + 10p means the 30-item test with 20 dichotomous items and 10 polytomous items.*

**TABLE 3:** Absolute bias and root mean squared difference for WLE, TWLE, and IWLE at 3 different levels of growth on 20d+10p.

20d+10p	Methods					
*N* = 1000	IWLE		WLE		TWLE	
θ_*2*_	Abs.Bias	RMSD	Abs.Bias	RMSD	Abs.Bias	RMSD
1	0.4219	1.9411	1.0146	3.7642	1.0438	3.7842
1	0.2559	1.5026	0.4351	2.5015	0.4801	2.5460
1	0.0907	1.0243	0.1419	1.4468	0.1536	1.4836
1	0.0215	0.6990	0.0296	0.6428	0.0399	0.7196
0.8	0.0145	0.6201	0.0050	0.5732	0.0225	0.6349
0.8	0.0078	0.5947	0.0071	0.5492	0.0101	0.6095
0.8	0.0188	0.5555	0.0144	0.5204	0.0194	0.5697
0.8	0.0055	0.5402	0.0073	0.4952	0.0075	0.5518
0.8	0.0054	0.5365	0.0042	0.5032	0.0100	0.5435
0.8	0.0283	0.5380	0.0236	0.4971	0.0276	0.5439
0.8	0.0056	0.5670	0.0023	0.5213	0.0059	0.5761
0.8	0.0301	0.6168	0.0197	0.5684	0.0330	0.6224
0.8	0.0726	0.7236	0.0504	0.7475	0.0779	0.8220
0.6	0.0782	0.9579	0.1165	1.2499	0.1224	1.3136
0.6	0.2395	1.4137	0.4164	2.3609	0.4538	2.3896
0.6	0.3946	2.2477	0.9091	4.3898	0.9462	4.3887
0.6	0.7397	2.8244	1.7386	5.9953	1.7629	5.9489

*20d + 10p means the 30-item test with 20 dichotomous items and 10 polytomous items.*

**TABLE 4:** Absolute bias and root mean squared difference for WLE, TWLE, and IWLE at 17 different levels of initial ability on 15d+15p.

15d+15p	Methods					
*N* = 1000	IWLE		WLE		TWLE	
θ_*1*_	Abs.Bias	RMSD	Abs.Bias	RMSD	Abs.Bias	RMSD
−4.0	0.4756	1.4673	0.8064	2.9131	0.8465	2.9195
−3.5	0.1589	0.8002	0.1603	1.2443	0.2009	1.2795
−3.0	0.0605	0.6537	0.0866	0.9311	0.0965	0.9520
−2.5	0.0104	0.4371	0.0167	0.4411	0.0152	0.4712
−2.0	0.0229	0.4076	0.0266	0.3855	0.0384	0.421
−1.5	0.0163	0.3677	0.0102	0.3535	0.0146	0.3791
−1.0	0.0035	0.3349	0.0057	0.3223	0.0039	0.3425
−0.5	0.0092	0.3433	0.0038	0.3295	0.0093	0.3509
0.0	0.0038	0.3336	0.0015	0.3168	0.0039	0.3375
0.5	0.0038	0.3334	0.0050	0.3199	0.0074	0.3365
1.0	0.0001	0.3306	0.0054	0.3111	0.0038	0.3398
1.5	0.0040	0.3578	0.0003	0.3333	0.0024	0.3553
2.0	0.0160	0.3776	0.0113	0.3611	0.0164	0.3796
2.5	0.0300	0.4917	0.0248	0.5686	0.0348	0.5867
3.0	0.1358	0.6881	0.1997	0.9829	0.1484	1.0048
3.5	0.2461	1.0279	0.2718	1.7932	0.3194	1.8233
4.0	0.4730	1.5333	0.8051	3.1026	0.8775	3.1494

*15d + 15p means the 30-item test with 15 dichotomous items and 15 polytomous items.*

**TABLE 5:** Absolute bias and root mean squared difference for WLE, TWLE, and IWLE at 3 different levels of growth on 15d+15p.

15d+15p	Methods					
*N* = 1000	IWLE		WLE		TWLE	
θ_*2*_	Abs.Bias	RMSD	Abs.Bias	RMSD	Abs.Bias	RMSD
1	0.3555	0.5993	0.7385	3.0097	0.7626	3.0257
1	0.0864	0.9536	0.1136	1.3520	0.1371	1.4041
1	0.0877	0.7725	0.1020	1.0090	0.1020	1.0338
1	0.0030	0.5857	0.0053	0.6040	0.0049	0.6358
0.8	0.0013	0.5067	0.0063	0.5133	0.0025	0.5528
0.8	0.0103	0.4933	0.0030	0.4738	0.0132	0.5085
0.8	0.0022	0.4669	0.0070	0.4513	0.0029	0.4735
0.8	0.0162	0.4728	0.0123	0.4462	0.0178	0.4820
0.8	0.0087	0.4572	0.0003	0.4324	0.0096	0.4603
0.8	0.0161	0.4787	0.0129	0.4531	0.0164	0.4796
0.8	0.0177	0.4941	0.0178	0.4640	0.0151	0.4906
0.8	0.0407	0.5626	0.0328	0.5328	0.0490	0.5632
0.8	0.0473	0.5864	0.0487	0.5805	0.0476	0.6206
0.6	0.0617	0.8184	0.0618	0.9583	0.0629	0.9855
0.6	0.1824	1.3755	0.3231	2.3313	0.3333	2.3686
0.6	0.3534	2.0572	0.8409	4.0178	0.8563	4.0486
0.6	0.5312	2.8113	1.4114	6.0129	1.4160	6.0353

*15d + 15p means the 30-item test with 15 dichotomous items and 15 polytomous items.*

**TABLE 6:** Absolute bias and root mean squared difference for WLE, TWLE, and IWLE at 17 different levels of initial ability for 10d + 20p.

10d+20p	Methods					
*N* = 1000	IWLE		WLE		TWLE	
θ_*1*_	Abs.Bias	RMSD	Abs.Bias	RMSD	Abs.Bias	RMSD
−4.0	0.4139	1.4081	0.7042	2.7417	0.8002	2.9845
−3.5	0.1748	0.8937	0.2045	1.4292	0.2356	1.5412
−3.0	0.0547	0.5415	0.0580	0.7017	0.0750	0.7492
−2.5	0.0108	0.4168	0.0155	0.4233	0.0223	0.4351
−2.0	0.0035	0.3677	0.0073	0.3540	0.0047	0.3691
−1.5	0.0020	0.3567	0.0023	0.3459	0.0038	0.3579
−1.0	0.0275	0.3360	0.0237	0.3253	0.0241	0.3421
−0.5	0.0211	0.3281	0.0145	0.3196	0.0200	0.3295
0	0.0039	0.3087	0.0040	0.2968	0.0047	0.3130
0.5	0.0010	0.3030	0.0007	0.2883	0.0018	0.3053
1.0	0.0089	0.2886	0.0054	0.2798	0.0115	0.2903
1.5	0.0066	0.3048	0.0000	0.2963	0.0210	0.3024
2.0	0.0081	0.3392	0.0073	0.3282	0.0109	0.3391
2.5	0.0182	0.3904	0.0234	0.3912	0.0327	0.4157
3.0	0.0205	0.5022	0.0288	0.5837	0.0436	0.5952
3.5	0.1616	0.8334	0.1687	1.3778	0.2042	1.3954
4.0	0.3306	1.2024	0.4565	2.3132	0.5022	2.3207

*10d + 20p means the 30-item test with 10 dichotomous items and 20 polytomous items.*

**TABLE 7:** Absolute bias and root mean squared difference for WLE, TWLE, and IWLE at 3 different levels of growth for 10d + 20p.

10d+20p	Methods					
*N* = 1000	IWLE		WLE		TWLE	
θ_*2*_	Abs.Bias	RMSD	Abs.Bias	RMSD	Abs.Bias	RMSD
1	0.3451	1.5350	0.6859	2.8275	0.7519	3.0801
1	0.1355	0.9881	0.1899	1.4906	0.2130	1.6003
1	0.0359	0.6451	0.0560	0.7772	0.0650	0.8253
1	0.0111	0.5061	0.0188	0.5386	0.0253	0.5580
0.8	0.0057	0.5151	0.0109	0.4972	0.0078	0.5181
0.8	0.0056	0.4737	0.0002	0.4611	0.0095	0.4753
0.8	0.0198	0.4510	0.0196	0.4408	0.0199	0.4593
0.8	0.0162	0.4532	0.0116	0.4398	0.0168	0.4536
0.8	0.0168	0.4385	0.0167	0.4201	0.0169	0.4428
0.8	0.0008	0.4142	0.0028	0.3979	0.0032	0.4152
0.8	0.0276	0.4301	0.0204	0.4293	0.0277	0.4389
0.8	0.0089	0.4325	0.0140	0.4250	0.0119	0.4349
0.8	0.0408	0.5412	0.0375	0.5347	0.0466	0.5510
0.6	0.0123	0.6007	0.0151	0.6038	0.0270	0.6342
0.6	0.1462	1.0715	0.2162	1.7098	0.2350	1.7319
0.6	0.3919	1.8900	0.8098	3.5369	0.8265	3.5700
0.6	0.5514	2.4670	1.3889	5.0994	1.4169	5.1159

*10d + 20p means the 30-item test with 10 dichotomous items and 20 polytomous items.*

Examining these results, the following general trends are observed. The absolute bias are all nearly to zero for three estimators when |*θ*_1_| < 2, or *θ*_2_ = 0.8, but IWLE has a considerably less absolute bias than the other two estimators when |*θ*_1_| > 2 or *θ*_2_ = 0.6 and 1. We note that in the 3 simulation scenarios the absolute bias of IWLE is slightly larger than that of WLE at some level of *θ*_1_ when |*θ*_1_| < 2, but is considerably smaller than that of WLE at the low and the high levels of ability. IWLE consistently displays the level of absolute bias that is smaller than that of TWLE, especially substantially smaller than that of TWLE at the low and the high levels of ability. In addition, the absolute bias of WLE is less than that of TWLE at the extremes of ability level. However, the changes are observed when the proportion of the dichotomous and polytomous items in mixed-type test is changed. With the number of polytomous items increased, the absolute bias produced by TWLE and WLE are more similar, even TWLE produces a little larger absolute bias than WLE at the extremes of ability level. The similar change patterns are also observed for RMSD produced by three estimators. The RMSD of IWLE is slightly larger than that of WLE at some level of *θ*_1_ when |*θ*_1_| < 2, but is considerably smaller than that of WLE and TWLE at the low and the high levels of ability.

**FIGURE 3:**
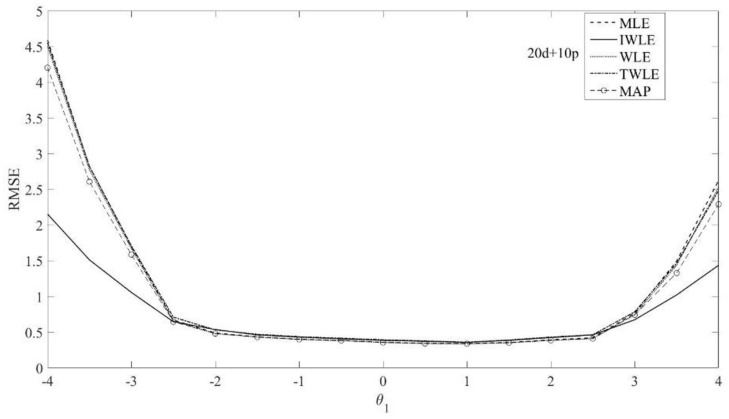
RMSE of the five *θ*_1_ estimation methods MLE, MAP, IWLE, WLE, and TWLE for 20d+10p.

**FIGURE 4:**
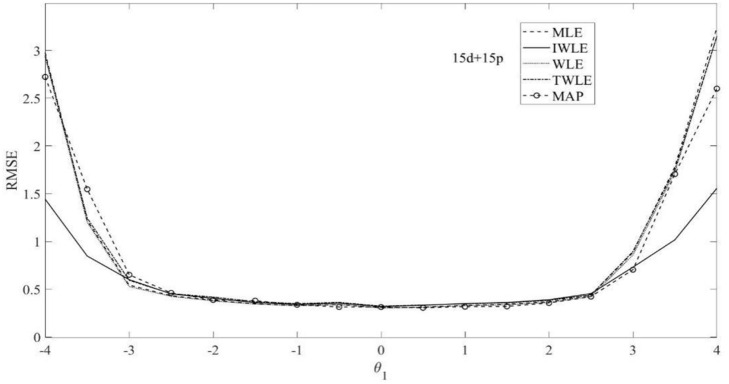
RMSE of the five *θ*_1_ estimation methods MLE, MAP, IWLE, WLE, and TWLE for 15d+15p.

**FIGURE 5:**
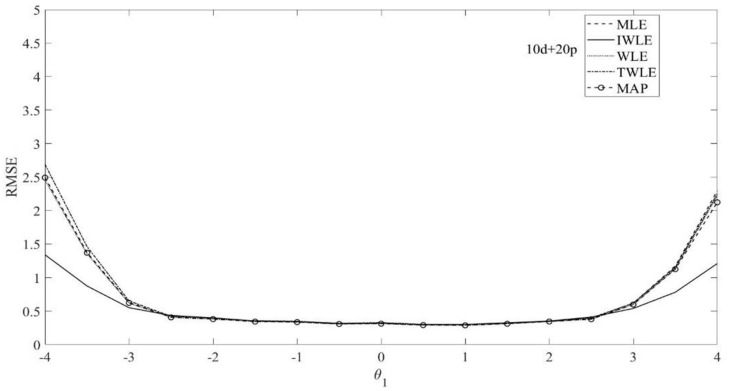
RMSE of the five *θ*_1_ estimation methods MLE, MAP, IWLE, WLE, and TWLE for 10d+20p.

**FIGURE 6:**
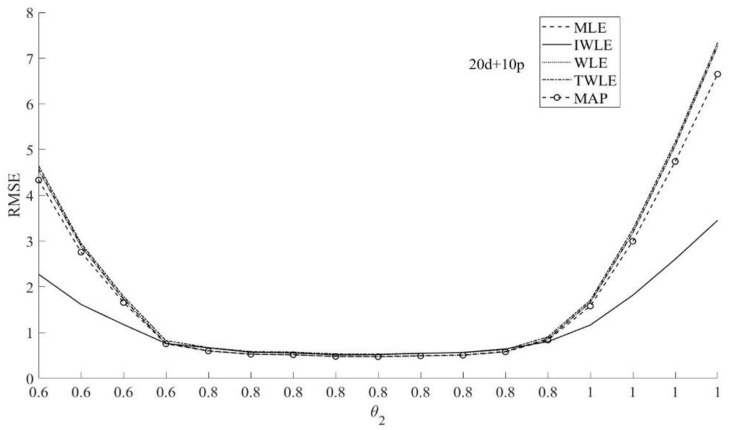
RMSE of the five *θ*_2_ estimation methods MLE, MAP, IWLE, WLE, and TWLE for 20d+10p.

**FIGURE 7:**
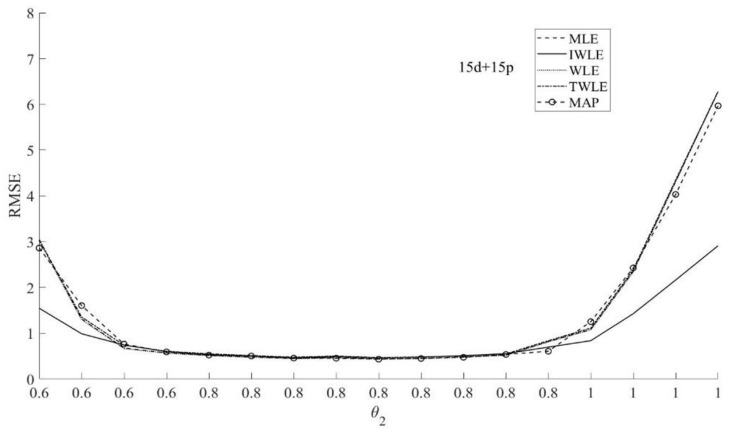
RMSE of the five *θ*_2_ estimation methods MLE, MAP, IWLE, WLE, and TWLE for 15d+15p.

**FIGURE 8:**
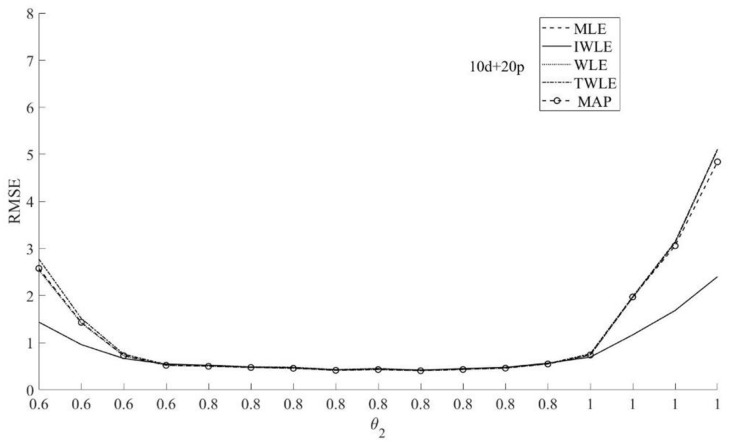
RMSE of the five *θ*_2_ estimation methods MLE, MAP, IWLE, WLE, and TWLE for 10d+20p.

To investigate the performance of the proposed IWLE method, an simulation study was conducted for the comparison of the five estimators: MLE, MAP [with a non-informative prior distribution *U*(4,4)] WLE, TWLE, and IWLE under the above simulation condition. Figures 3–8 show the results of RMSE calculated from 30-item test in the following simulation scenarios:

(1). 30-item test includes 20 dichotomous items and 10 polytomous items (20d + 10p).
(2). 30-item test includes 15 dichotomous items and 15 polytomous items (15d + 15p).
(3). 30-item test includes 10 dichotomous items and 20 polytomous items (10d + 20p).

The RMSE presented in Figures 3–5 show that among the five *θ*_1_ estimation methods, IWLE has a slight large RMSE when |*θ*_1_| < 2, but is considerably smaller than that of MLE, MAP, WLE and TWLE at extreme levels of the latent trait. The RMSE of WLE is very similar to that of MLE and TWLE. EAP has lower RMSE than MLE, WLE, TWLE, and IWLE in the middle of the ability range because of the shrinkage. The RMSE plotted in Figures 6–8 shows the similar change patterns for *θ*_2_.

The proposed IWLE method outperforms the MLE, MAP, WLE and TWLE in terms of controlling the absolute bias, RMSE, and RMSD at the low and the high levels of ability, but has a slight large RMSE and RMSD in the middle range of the ability scale.

In general, test length had a dramatic impact on the relative performance of the five estimators. We can observe the strongest differences between the five estimators are obtained when the test length is short. The absolute bias, RMSE, and RMSD of five estimation methods have a slightly decrease with the length of test increased. The proportion of dichotomous and polytomous items in a mixed-format test appears to affect the absolute bias, RMSE, and RMSD of five estimation methods.

## Simulation Study 2

When we only care about the ability of the examinee without considering the ability growth at multiple time points, the unidimensional IRT models are the focus of many educational psychometrists. In fact, our IWLE method can’t only be used to analyze multidimensional IRT models, but also can be implemented for unidimensional IRT models. In this simulation study, we evaluate the accuracy of the IWLE method in the unidimensional models.

The proposed IWLE method is applied to the unidimensional IRT models for mixed-format test that is the combination of the two-parameter logistic model and the partial-credit model. We consider the following item-weighted likelihood function:

$$IWL\left(\theta |\text{U}\right)=IWL_{d}\left(\theta |\text{U}\right)\cdot IWL_{p}\left(\theta |\text{U}\right),$$

where

$$IWL_{d}\left(\theta |\text{U}\right)=\prod_{i=1}^{k}{\left\{P_{i}{\left(\theta \right)}^{u_{i}}\cdot Q_{i}{\left(\theta \right)}^{1-u_{i}}\right\}}^{w_{i}\left(\theta \right)},$$

and

$$IWL_{p}\left(\theta |\text{U}\right)=\prod_{i=k+1}^{24^{n}}\prod_{j=1}^{h};{\left\{P_{ij}{\left(\theta \right)}^{u_{ij}}\right\}}^{w_{i}\left(\theta \right)},$$

*P*_*i*_(θ) is determined by dichotomously scored items; *P*_*ik*_(θ) is determined by polytomously scored items. Here the weight *w*_*i*_(θ) assigned to item *i* is defined as equation 4, and $\sum_{i=1}^{n}w_{i}\left(\theta \right)=1.$ The 3 levels of test length (10 items, 30 items and 60 items) and the 3 levels of proportion of dichotomous and polytomous items (λ = 2,1,0.5) were selected. The item parameters were generated similar to simulation 1, and 17 equally spaced *θ*_1_ values were considered, ranging from –4.0 to 4.0 in increments of 0.5.

**FIGURE 9:**
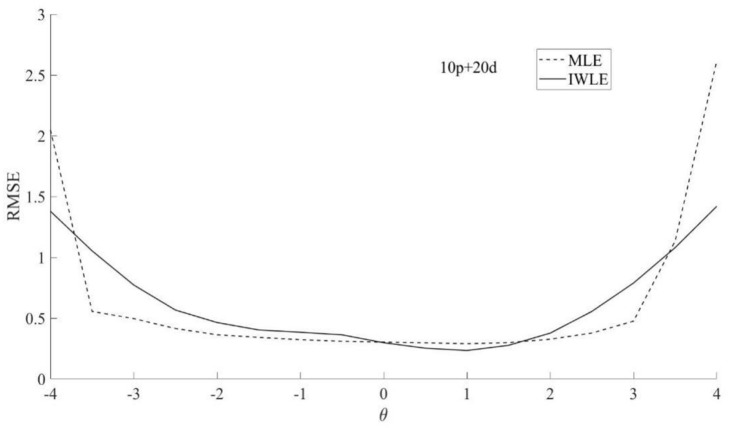
RMSE of the two θ estimation methods MLE and IWLE for 10p+20d.

**FIGURE 10:**
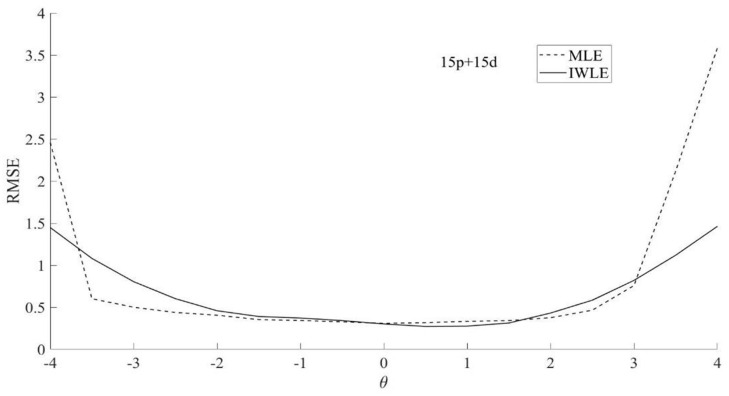
RMSE of the two θ estimation methods MLE and IWLE for 15p+15d.

**FIGURE 11:**
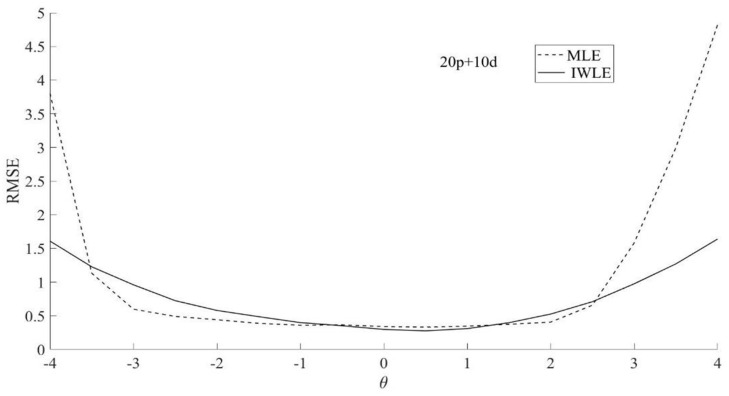
RMSE of the two θ estimation methods MLE and IWLE for 20p+10d.

The simulation results of three test lengths show similar trends. The proposed IWLE method outperforms the MLE in terms of the absolute bias, RMSE and RMSD at the low and high levels of ability. However, the IWLE has a slight large absolute bias, RMSE and RMSD in the middle range of the ability scale compared with the MLE. Figures 9–11 show the results of RMSE calculated from 30-item test. According to the simulation results, we find that the IWLE can also be applied to the general unidimensional IRT models for tests composed of both dichotomous and polytomous items.

## Discussion and Conclusion

In this study, an improved IWLE procedure that incorporates item weights in likelihood functions for the ability parameter estimation is proposed. The weights may be “adaptive” in the sense that they are allowed to be estimated with the ability level and individual test items. We assign different weights to different items in accordance with the amount of the information an item provides at a certain latent trait level. Using the information ratio of each item to the test, the weights of items are determined. We also give the rigorous derivations for asymptotic properties and the bias of IWL estimators. The results from the simulation study clearly demonstrate that the proposed IWLE method outperforms the usual, MLE, MAP, WLE and TWLE in terms of controlling absolute bias, RMSE, and RMSD especially at low and high ability levels. Latent trait estimation is one of the most important components in IRT, but when an examinee scores high (or low) in a test, we known that the examinee is high (or low) on the trait but we do not have a very precise estimate of how high (or low). It could be considerably higher (or lower) than the test instrument’ scale reaches. In the case, improving latent trait estimation especially at extreme levels of ability scale is worthy of attention.

Improving latent trait estimation is always important in longitudinal survey assessments, such as the Early Childhood Longitudinal Study (ECLS) and the PISA (von Davier and Xu, 2011), which aims at tracking growth of a representative sample of the target population over time. The proposed weighting scheme also can be applied in the general unidimensional item response models. Other issues should be further explored. First, the proposed weighting scheme could be generalized to other application settings where latent ability needs to be estimated for each person such as computerized adaptive testing (CAT). Second, although the Rasch model and the PCM are commonly used in practical tests, there are other more general item response models, for instance the three-parameter logistic (3PL) model and the generalized partial credit model. Therefore, it is worth studying the extension of the IWLE to these more complex models, with different test lengths and sample sizes. Third, more than two occasions can be considered in longitudinal study, so the proposed weighting method can be generalized to deal with more general situations. Finally, the proposed IWLE method can be extended to multidimensional longitudinal IRT model.

From a practical point of view, we would not use a test that is way too difficult or way too easy items. This is because each item should have a certain discrimination to distinguish the examinees with different ability levels. In fact, the reliability and validity of the test items are pre-calibrated before the actual assessment. When the examinees answer the pre-calibrated test, some examinees answer all items correctly while others do not answer all items correctly. In this case, the extreme ability estimator will occur. Thus, the extreme ability occur because there are large differences between examinees’ abilities rather than items being too difficult or too easy (the test items are pre-calibrated, reliable and valid). In addition, the examinees were obtained through a multistage stratified sample in the actual assessment. In the first stage, the sampling population is classified according to district, and schools are selected at random. In the second stage, students are selected at random from each school. Therefore, in this case, there are some extreme cases of the examinees’ ability. For example, some examinees with high abilities answer all the items correctly, or some examinees with low abilities answered all the items incorrectly. Traditional methods (WLE and TWLE) fail to estimate these extreme abilities. However, our IWLE method is more accurate in estimating these extreme abilities. This is the main advantage of our item-weighted scheme.

## Data Availability Statement

The raw data supporting the conclusion of this article will be made available by the authors, without undue reservation.

## Author Contributions

XX completed the writing of the article. XX and JL provided key technical support. JZ provided original thoughts and article revisions. All authors contributed to the article and approved the submitted version.

## Conflict of Interest

The authors declare that the research was conducted in the absence of any commercial or financial relationships that could be construed as a potential conflict of interest.

## Publisher’s Note

All claims expressed in this article are solely those of the authors and do not necessarily represent those of their affiliated organizations, or those of the publisher, the editors and the reviewers. Any product that may be evaluated in this article, or claim that may be made by its manufacturer, is not guaranteed or endorsed by the publisher.
