# Supplementary material for: Item-Weighted Likelihood Method for Measuring Growth in Longitudinal Study With Tests Composed of Both Dichotomous and Polytomous Items
Source: Front Psychol. 2021 Jul 27;12:580015. doi: 10.3389/fpsyg.2021.580015 (PMC8353132; doi:10.3389/fpsyg.2021.580015)
Supplement: Supplementary file 1 [file Presentation_1.pdf]

Supplementary material A

Details of Item-Weighted likelihood Estimation Using the Newton-Raphson Algorithm

We can obtain the item-weighted likelihood (IWL) estimator by solving Equation 16,

$$\begin{cases} \frac{\partial \log IWL(\boldsymbol{\theta}|\mathbf{U})}{\partial \theta_1} = 0 \\ \frac{\partial \log IWL(\boldsymbol{\theta}|\mathbf{U})}{\partial \theta_2} = 0 \end{cases}, \tag{16}$$

where $IWL(\boldsymbol{\theta}|\mathbf{U}) = IWL_d(\boldsymbol{\theta}|\mathbf{U}) \cdot IWL_p(\boldsymbol{\theta}|\mathbf{U})$ denotes the weighted likelihood function of the mixed-type model, as defined in Equation 9.

The solution of Equation 16 can be obtained using the Fisher scoring method as follows:

$$\begin{bmatrix} \hat{\theta}_1 \\ \hat{\theta}_2 \end{bmatrix}_{(t+1)} = \begin{bmatrix} \hat{\theta}_1 \\ \hat{\theta}_2 \end{bmatrix}_{(t)} - \begin{bmatrix} \frac{\partial^2 \log IWL}{\partial \theta_1^2} & \frac{\partial^2 \log IWL}{\partial \theta_1 \partial \theta_2} \\ \frac{\partial^2 \log IWL}{\partial \theta_2 \partial \theta_1} & \frac{\partial^2 \log IWL}{\partial \theta_2^2} \end{bmatrix}^{-1} \begin{bmatrix} \frac{\partial \log IWL}{\partial \theta_1} \\ \frac{\partial \log IWL}{\partial \theta_2} \end{bmatrix}_{(t)}, \tag{17}$$

where $\log IWL(\boldsymbol{\theta}|\mathbf{U}) = \log IWL_d(\boldsymbol{\theta}|\mathbf{U}) + \log IWL_p(\boldsymbol{\theta}|\mathbf{U})$. For convenience, we let

$IWL = IWL(\boldsymbol{\theta}|\mathbf{U})$,$P_{i1} = P_{i1}(\theta_1)$,$P_{i2} = P_{i2}(\theta_1,\theta_2)$,$Q_{ij} = 1 - P_{ij}$,$P_{ij1} = P_{ij1}(\theta_1)$,$P_{ij2} = P_{ij2}(\theta_1,\theta_2)$, $j = 1,2$, and note that $\boldsymbol{\theta}$,θ_1 and θ_2 are not shown in the following derivations.

Thus,

$\log IWL_d = \sum_{i=1}^{k} w_i[u_i \log P_{i1} + (1 - u_i)\log Q_{i1}] + \sum_{i=n_1+1}^{m} w_i[v_i \log P_{i2} + (1 - v_i)\log Q_{i2}]$,

$$\log IWL_p = \sum_{i=k+1}^{n_1} w_i \left(\sum_{j=1}^{h} u_{ij} \log P_{ij1}\right) + \sum_{i=m+1}^{n} w_i \left(\sum_{j=1}^{h} v_{ij} \log P_{ij2}\right),$$

where $w_i = w_i(\boldsymbol{\theta})$ $i = 1,2,...,n$, are defined in Equation 12. We have

$$\frac{\partial w_i}{\partial \theta_i} = \frac{\frac{\partial I_i}{\partial \theta_i} I - I_i \frac{\partial I}{\partial \theta_i}}{I^2}, i = 1,2, \tag{18}$$

$$\frac{\partial^2 w_i}{\partial \theta_i^2} = \frac{I\left(\frac{\partial^2 I_i}{\partial \theta_i^2} I - I_i \frac{\partial^2 I}{\partial \theta_i^2}\right) - 2\frac{\partial I}{\partial \theta_i}\left(\frac{\partial I_i}{\partial \theta_i} I - I_i \frac{\partial I}{\partial \theta_i}\right)}{I^3}, i = 1,2, \tag{19}$$

$$\frac{\partial^2 w_i}{\partial \theta_2 \partial \theta_1} = \frac{I\left(\frac{\partial^2 I_i}{\partial \theta_2 \partial \theta_1} \cdot I + \frac{\partial I_i}{\partial \theta_1} \cdot \frac{\partial I}{\partial \theta_2} - \frac{\partial I}{\partial \theta_1} \cdot \frac{\partial I_i}{\partial \theta_2} - I_i \cdot \frac{\partial^2 I}{\partial \theta_2 \partial \theta_1}\right) - 2\frac{\partial I}{\partial \theta_2}\left(\frac{\partial I_i}{\partial \theta_1} \cdot I - I_i \cdot \frac{\partial I}{\partial \theta_1}\right)}{I^3}, \tag{20}$$

$$\frac{\partial^2 w_i}{\partial \theta_1 \partial \theta_2} = \frac{\partial^2 w_i}{\partial \theta_2 \partial \theta_1}. \tag{21}$$

The test information function I is composed of two parts:

$$I = I_d + I_p,$$

where

$$I_d = \sum_{i=1}^{k} P_{i1} Q_{i1} + \sum_{i=n_1+1}^{m} P_{i2} Q_{i2}$$

and

$$I_p = \sum_{i=k+1}^{n_1} \left[\sum_{j=1}^{h} j^2 P_{ij1} - \left(\sum_{j=1}^{h} j P_{ij1}\right)^2\right] + \sum_{i=m+1}^{n} \left[\sum_{j=1}^{h} j^2 P_{ij2} - \left(\sum_{j=1}^{h} j P_{ij2}\right)^2\right]$$

are the dichotomous part and the polytomous part, respectively.

Taking the first and the second partial derivatives of I_d and I_p about θ_1 and θ_2, respectively, we have

$$\frac{\partial I_d}{\partial \theta_1} = \sum_{i=1}^{k} P_{i1}Q_{i1}(Q_{i1} - P_{i1}) + \sum_{i=\, n_1\, +1}^{m} P_{i2}Q_{i2}(Q_{i2} - P_{i2}),$$

$$\frac{\partial I_p}{\partial \theta_1} = \sum_{i=k+1}^{n_1} \left(w_{13} - 3w_{11}w_{12} + 2w_{11}^3\right) + \sum_{i=m+1}^{n} \left(w_{23} - 3w_{21}w_{22} + 2w_{21}^3\right),$$

$$\frac{\partial^2 I_d}{\partial \theta_1^2} = \sum_{i=1}^{k} \left(P_{i1}Q_{il}^3 - 4P_{i1}^2Q_{i1}^2 + P_{i1}^3Q_{i1}\right) + \sum_{i=\, n_1\, +1}^{m} \left(P_{i2}Q_{i2}^3 - 4P_{i2}^2Q_{i2}^2 + P_{i2}^3Q_{i2}\right),$$

$$\frac{\partial^2 I_p}{\partial \theta_1^2} = \sum_{i=k+1}^{n_1} \left(w_{14} - 4w_{11}w_{13} - 3w_{12}^2 + 12w_{11}^2w_{12} - 6w_{11}^4\right) + \sum_{i=m+1}^{n} (w_{24} - 4w_{21}w_{23} - 3w_{22}^2 + 12w_{21}^2w_{22} - 6w_{21}^4),$$

$$\frac{dI_d}{d\theta_2} = \sum_{i=\, n_1\, +1}^{m} P_{i2}Q_{i2}(Q_{i2} - P_{i2}),$$

$$\frac{\partial I_p}{\partial \theta_2} = \sum_{i=m+1}^{n} \left(w_{23} - 3w_{21}w_{22} + 2w_{21}^3\right),$$

$$\frac{\partial^2 I_d}{\partial \theta_2^2} = \sum_{i=\, n_1\, +1}^{m} \left(P_{i2}Q_{i2}^3 - 4P_{i2}^2Q_{i2}^2 + P_{i2}^3Q_{i2}\right),$$

$$\frac{\partial^2 I_p}{\partial \theta_2^2} = \sum_{i=m+1}^{n} \left(w_{24} - 4w_{21}w_{23} - 3w_{22}^2 + 12w_{21}^2w_{22} - 6w_{21}^4\right),$$

$$\frac{\partial^2 I_d}{\partial \theta_1 \partial \theta_2} = \frac{\partial^2 I_d}{\partial \theta_2 \partial \theta_1} = \sum_{i=\, n_1\, +1}^{m} \left(12P_{i2}^3 - 6P_{i2}^4 - 7P_{i2}^2 + P_{i2}\right),$$

$$\frac{\partial^2 I_p}{\partial \theta_1 \partial \theta_2} = \frac{\partial^2 I_p}{\partial \theta_2 \partial \theta_1} = \sum_{i=m+1}^{n} \left(w_{24} - 4w_{21}w_{23} - 3w_{22}^2 + 12w_{21}^2w_{22} - 6w_{21}^4\right),$$

where $w_{st} = \sum_{j=1}^{h} j^t P_{ijs}$,$s = 1,2, t = 1,2,3,4, i = 1,2,\ldots,n$.

Substituting

$$\frac{\partial I}{\partial \theta_i} = \frac{\partial I_d}{\partial \theta_i} + \frac{\partial I_p}{\partial \theta_i}, \frac{\partial^2 I}{\partial \theta_i^2} = \frac{\partial^2 I_d}{\partial \theta_i^2} + \frac{\partial^2 I_p}{\partial \theta_i^2}, i = 1,2,$$

$$\frac{\partial^2 I}{\partial \theta_1 \partial \theta_2} = \frac{\partial^2 I}{\partial \theta_2 \partial \theta_1} = \frac{\partial^2 I_d}{\partial \theta_2 \partial \theta_1} + \frac{\partial^2 I_p}{\partial \theta_2 \partial \theta_1},$$

I, I_d, I_p into Equations 18 to 21, we obtain $\frac{\partial w_i}{\partial \theta_i}, \frac{\partial^2 w_i}{\partial \theta_i^2}, i = 1,2,$ and $\frac{\partial^2 w_i}{\partial \theta_2 \partial \theta_1}$.

Taking the first partial derivative of log IWL about θ_1 and θ_2 yields

$$\frac{\partial \log IWL}{\partial \theta_1} = \frac{\partial \log IWL_d}{\partial \theta_1} + \frac{\partial \log IWL_p}{\partial \theta_1}, \tag{22}$$

where

$$\frac{\partial \log IWL_d}{\partial \theta_1} = \sum_{i=1}^{k} \frac{\partial w_i}{\partial \theta_1}[u_i \log P_{i1} + (1-u_i)\log Q_{i1}] + \sum_{i=1}^{k} w_i(u_i - P_{i1}) + \sum_{i=n_1+1}^{m} \frac{\partial w_i}{\partial \theta_1}[v_i \log P_{i2} + (1-v_i)\log Q_{i2}] + \sum_{i=n_1+1}^{m} w_i(v_i - P_{i2}), \tag{23}$$

and

$$\frac{\partial \log IWL_p}{\partial \theta_1} = \sum_{i=k+1}^{n_1} \frac{\partial w_i}{\partial \theta_1}\left(\sum_{j=1}^{h} u_{ij} \log P_{ij1}\right) + \sum_{i=k+1}^{n_1} w_i \left[\sum_{j=1}^{h} u_{ij}\left(j - \sum_{j=1}^{h} jP_{ij1}\right)\right] + \sum_{i=m+1}^{n} \frac{\partial w_i}{\partial \theta_1}\left(\sum_{j=1}^{h} v_{ij} \log P_{ij2}\right) + \sum_{i=m+1}^{n} w_i \left[\sum_{j=1}^{h} v_{ij}\left(j - \sum_{j=1}^{h} jP_{ij2}\right)\right]. \tag{24}$$

And

$$\frac{\partial \log IWL}{\partial \theta_2} = \frac{\partial \log IWL_d}{\partial \theta_2} + \frac{\partial \log IWL_p}{\partial \theta_2}$$

$$= \sum_{i=n_1+1}^{m} \frac{\partial w_i}{\partial \theta_2} [v_i \log P_{i2} + (1 - v_i) \log Q_{i2}] + \sum_{i=n_1+1}^{m} w_i (v_i - P_{i2})$$

$$+ \sum_{i=m+1}^{n} \frac{\partial w_i}{\partial \theta_2} \left(\sum_{j=1}^{h} v_{ij} \log P_{ij2} \right) + \sum_{i=m+1}^{n} w_i \left[\sum_{j=1}^{h} v_{ij} \left(j - \sum_{j=1}^{h} j P_{ij2} \right) \right]. \quad (25)$$

Taking the second partial derivative of log IWL about θ_1 and θ_2, and then take

expectations for them yields

$$E \frac{\partial^2 \log IWL}{\partial \theta_1^2} = E \frac{\partial^2 \log IWL_d}{\partial \theta_1^2} + E \frac{\partial^2 \log IWL_p}{\partial \theta_1^2}, \quad (26)$$

where

$$E \frac{\partial^2 \log IWL_d}{\partial \theta_1^2} = \sum_{i=1}^{k} \frac{\partial^2 w_i}{\partial \theta_1^2} [\mathrm{P}_{i1} \log P_{i1} + (1 - P_{i1}) \log Q_{i1}] - \sum_{i=1}^{k} w_i P_{i1} Q_{i1}$$

$$+ \sum_{i=n_1+1}^{m} \frac{\partial^2 w_i}{\partial \theta^2} [\mathrm{P}_{i2} \log P_{i2} + (1 - P_{i2}) \log Q_{i2}] - \sum_{i=n_1+1}^{m} w_i P_{i2} Q_{i2} \quad ,$$

(27)

and

$$E \frac{\partial^2 \log IWL_p}{\partial \theta_1^2}$$

$$= \sum_{i=k+1}^{n_1} \frac{\partial^2 w_i}{\partial \theta_1^2} \left(\sum_{j=1}^{h} P_{ij1} \log P_{ij1} \right) - \sum_{i=k+1}^{n_1} w_i \left[\sum_{j=1}^{h} j^2 P_{ij1} - \left(\sum_{j=1}^{h} j P_{ij1} \right)^2 \right]$$

$$+ \sum_{i=m+1}^{n} \frac{\partial^2 w_i}{\partial \theta_1^2} \left(\sum_{j=1}^{h} P_{ij2} \log P_{ij2} \right) - \sum_{i=m+1}^{n} w_i \left[\sum_{j=1}^{h} j^2 P_{ij2} - \left(\sum_{j=1}^{h} j P_{ij2} \right)^2 \right]. \quad (28)$$

$$E\frac{\partial^2 \log IWL}{\partial\theta_2^2} = E\frac{\partial^2 \log IWL_d}{\partial\theta_2^2} + E\frac{\partial^2 \log IWL_p}{\partial\theta_2^2}, \tag{29}$$

$$E\frac{\partial^2 \log IWL_d}{\partial\theta_2^2} = \sum_{i=n_1+1}^{m} \frac{\partial^2 w_i}{\partial\theta_2^2}[\mathrm{P}_{i2}\log P_{i2} + (1-P_{i2})\log Q_{i2}] - \sum_{i=n_1+1}^{m} w_i P_{i2} Q_{i2}, \tag{30}$$

and

$$E\frac{\partial^2 \log IWL_p}{\partial\theta_2^2} = \sum_{i=m+1}^{n} \frac{\partial^2 w_i}{\partial\theta_2^2}\left(\sum_{j=1}^{h} P_{ij2}\log P_{ij2}\right) - \sum_{i=m+1}^{n} w_i \left[\sum_{j=1}^{h} j^2 P_{ij2} - \left(\sum_{j=1}^{h} jP_{ij2}\right)^2\right]. \tag{31}$$

$$E\frac{\partial^2 \log IWL}{\partial\theta_1\partial\theta_2} = E\frac{\partial^2 \log IWL_d}{\partial\theta_1\partial\theta_2} + E\frac{\partial^2 \log IWL_p}{\partial\theta_1\partial\theta_2}, \tag{32}$$

Where

$$E\frac{\partial^2 \log IWL_d}{\partial\theta_1\partial\theta_2} = \sum_{i=n_1+1}^{m} \frac{\partial^2 w_i}{\partial\theta_1\partial\theta_2}[P_{i2}\log P_{i2} + (1-P_{i2})\log Q_{i2}] - \sum_{i=n_1+1}^{m} w_i P_{i2} Q_{i2}, \tag{33}$$

$$E\frac{\partial^2 \log IWL_p}{\partial\theta_1\partial\theta_2} = \sum_{i=m+1}^{n} \frac{\partial^2 w_i}{\partial\theta_1\partial\theta_2}\left(\sum_{j=1}^{h} P_{ij2}\log P_{ij2}\right) - \sum_{i=m+1}^{n} w_i \left[\sum_{j=1}^{h} j^2 P_{ij2} - \left(\sum_{j=1}^{h} jP_{ij2}\right)^2\right]. \tag{34}$$

And

$$E\frac{\partial^2 \log IWL}{\partial\theta_1\partial\theta_2} = E\frac{\partial^2 \log IWL}{\partial\theta_2\partial\theta_1}. \tag{35}$$

Substituting $\frac{\partial w_i}{\partial\theta_i}$, $\frac{\partial^2 w_i}{\partial\theta_i^2}$, and $\frac{\partial^2 w_i}{\partial\theta_2\partial\theta_1}$ into Equations 22 to 35, we obtain Equation 17.

Supplementary material B

Detailed derivations for asymptotic properties and the bias of IWL estimators.

We deal with the more general case where the test composed of m dichotomous items and $n-m$ polytomous items, P_i is the three-parameter logistic function and P_{ij} is the item response category function of GPCM. The following is the item-weighted likelihood function:

$$IWL(\boldsymbol{\theta}|\mathbf{U}) = \prod_{i=1}^{n} f_i^{w_i} = \prod_{i=1}^{m} \left(P_i^{u_i} Q_i^{1-u_i}\right)^{w_i} \cdot \prod_{i=m+1}^{n} \left(\prod_{j=1}^{h} P_{ij}^{u_{ij}}\right)^{w_i},$$

where

$$f_i = \begin{cases} P_i^{u_i} Q_i^{1-u_i}, & 1 \le i \le m \\ \prod_{j=1}^{h} P_{ij}^{u_{ij}}, & m+1 \le i \le n \end{cases},$$

U is a response vector, and the weights w_i are constants which are determined at a certain ability level (e.g. $w_i = w_i(\hat{\boldsymbol{\theta}}), \hat{\boldsymbol{\theta}}$ is an estimator of $\boldsymbol{\theta}$). We assume that (a) $\boldsymbol{\theta} = (\theta_1, ..., \theta_k)$ lies in a k-dimensional bounded interval Ω. (b) The item parameters a_i, b_i are bounded and c_i is bounded away from 1, thus P_i and Q_i are bounded away from 0 and 1. (c) For asymptotic considerations n is considered to be incremented with replications of all of the original n experiments. With these assumptions the following conditions are satisfied which similar to that of Bradley and Gart (1962), we can obtain consistency and asymptotic normality of IWL estimators by these conditions.

Condition I:

I (i). For almost all $u_i \in \{0,1\}$ and $u_{ij} \in \{0,1\}$ and for all $\boldsymbol{\theta} \in \Omega$,

$$\frac{\partial \ln f_i}{\partial \theta_r}, \frac{\partial^2 \ln f_i}{\partial \theta_r \partial \theta_s}, \text{ and } \frac{\partial^3 \ln f_i}{\partial \theta_r \partial \theta_s \partial \theta_t}$$

exist for $r,s,t = 1,\ldots,k; i = 1,\ldots,n$.

I (ii). For all f_i that are probability functions,

$$\sum_u w_i \frac{\partial f_i}{\partial \theta_r}, \text{ and } \sum_u w_i \frac{\partial^2 f_i}{\partial \theta_r \partial \theta_s}$$

converge uniformly for all $\boldsymbol{\theta} \in \Omega$, and

$$\left| \frac{\partial^3 \ln f_i}{\partial \theta_r \partial \theta_s \partial \theta_t} \right| < H_{irst}(u_i),$$

where $\sum_{u_i} H_{irst}(u_i) f_i < M_i$, for all $\boldsymbol{\theta} \in \Omega, (r,s,t = 1,\ldots,k; i = 1,\ldots,n)$ and the M_i are finite positive constants. These conditions will permit the interchange of order differentiation and summation.

Condition II:

II (i). For $f_i, (i = 1,\ldots,n)$,

$$\sum_{i=1}^{n} \sum_{D_{1i}} f_i = o(1),$$

where D_{1i} is defined by the inequality $|w_i \cdot \partial \ln f_i / \partial \theta_r| > n$, and

$$\sum_{i=1}^{n} \sum_{D_{2i}} w_i^2 \left(\frac{\partial \ln f_i}{\partial \theta_r} \right)^2 f_i = o(n^2) \ (r = 1,\ldots,k),$$

where D_{2i} is defined by the inequality $|w_i \cdot \partial \ln f_i / \partial\theta_r| < n$.

II (ii). For f_i,$(i = 1,\ldots,n)$,

$$\sum_{i=1}^{n} \sum_{D_{3i}} f_i = o(1),$$

where D_{3i} is defined by the inequality $|w_i \cdot \partial^2 \ln f_i / \partial\theta_r \partial\theta_s| > n$, and

$$\sum_{i=1}^{n} \sum_{D_{4i}} w_i^2 \left(\frac{\partial^2 \ln f_i}{\partial\theta_r \partial\theta_s}\right)^2 f_i = o(n^2) \; (r = 1,\ldots,k),$$

where D_{4i} is defined by the inequality $|w_i \cdot \partial^2 \ln f_i / \partial\theta_r \partial\theta_s| < n$, and

$$\lim_{n\to\infty} \frac{1}{n} \sum_{i=1}^{n} \sum_{D_{4i}} w_i^2 \left(-\frac{\partial^2 \ln f_i}{\partial\theta_r \partial\theta_s}\right) f_i = \lim_{n\to\infty} \frac{1}{n} \sum_{i=1}^{n} w_i^2 E\left(\frac{\partial \ln f_i}{\partial\theta_r} \cdot \frac{\partial \ln f_i}{\partial\theta_s}\right)$$
$$= J_{rs}(\boldsymbol{\theta}) \; (r,s = 1,\ldots,k)$$

exist. $J = (J_{rs}(\boldsymbol{\theta}))$ is positive define with finite determinant.

II (iii). For f_i,$(i = 1,\ldots,n)$,

$$\sum_{i=1}^{n} \sum_{D_{ji}} f_i = o(1),$$

where D_{5i} is defined by the inequality $H_{irst}(u_i) > n$,

$$\sum_{i=1}^{n} \sum_{D_{6i}} H_{irst}^2(u_i) f_i = o(n^2),$$

where D_{6i} is defined by the inequality $H_{irst}(u_i) < n$, and $\frac{1}{n}\sum_{i=1}^{n} M_i < M$, M is a finite

positive constant.

III. $\lim_{n\to\infty}\frac{1}{n}\sum_{i=1}^{n}\sum_{D_{7i}}\sum_{r=1}^{k}w_i^2\left(\frac{\partial \ln f_i}{\partial\theta_r}\right)^2 f_i = 0,$

where D_{7i} is defined by the inequality $\left[\sum_{r=1}^{k}w_i^2\left(\frac{\partial \ln f_i}{\partial\theta_r}\right)^2\right]^{1/2} > \varepsilon\sqrt{n}$ for every $\varepsilon > 0$.

Theorem 1. Under Condition I and II, if $\hat{\boldsymbol{\theta}}$ represents a solution to the weighted likelihood equation for $\boldsymbol{\theta}$ and if $\boldsymbol{\theta}^0$ represents the true value of $\boldsymbol{\theta}$, then as $n\to\infty$,$\hat{\boldsymbol{\theta}}$ is a consistent estimator of $\boldsymbol{\theta}^0$.

Proof. Let $\boldsymbol{\theta}^0$ is an inner point of Ω. Consider the following Taylor expansion:

$$\frac{\partial \ln f_i^{w_i}}{\partial\theta_r} = \left(\frac{\partial \ln f_i^{w_i}}{\partial\theta_r}\right)_{\theta=\theta^0} + \sum (\theta_s-\theta_s^0)\left(\frac{\partial^2 \ln f_i^{w_i}}{\partial\theta_r\partial\theta_s}\right)_{\theta=\theta^0} + \frac{1}{2}\sum_{s,t=1}^{k}(\theta_s-\theta_s^0)(\theta_t-\theta_t^0)\left(\frac{\partial^3 \ln f_i^{w_i}}{\partial\theta_r\partial\theta_s\partial\theta_t}\right)_{\theta=\theta'}, \quad (36)$$

where $\boldsymbol{\theta}' = \boldsymbol{\theta}'(u)$ is a value depending on u, but for all u, lying inside the hyper-cell, of which the vector $\boldsymbol{\theta}-\boldsymbol{\theta}^{\mathbf{0}}$ is the diagonal. Multiplying both sides by $\frac{1}{n}$ and summing the corresponding expressions for $u_i's$ over $i = 1,...,n$, we may rewrite Equation 36 as

$$L_r(\boldsymbol{\theta}) = L_r(\boldsymbol{\theta}^0) - \sum_{s=1}^{k}\delta_s L_{rs}(\boldsymbol{\theta}^0) + \frac{1}{2}\sum_{s,t=1}^{k}\delta_s\delta_t L_{rst}\ (r = 1,...,k), \quad (37)$$

where

$$\delta_s = \theta_s - \theta^0,$$

$$L_r(\boldsymbol{\theta}) = \frac{1}{n}\sum_{i=1}^{n} \frac{\partial \ln f_i^{w_i}}{\partial \theta_r} = \frac{1}{n}\sum_{i=1}^{n} w_i \frac{\partial \ln f_i}{\partial \theta_r} = \frac{1}{n}\frac{\partial \ln IWL}{\partial \theta_r},$$

$$L_{rs}(\boldsymbol{\theta}) = -\frac{1}{n}\sum_{i=1}^{n} \frac{\partial^2 \ln f_i^{w_i}}{\partial \theta_r \partial \theta_s} = -\frac{1}{n}\sum_{i=1}^{n} w_i \frac{\partial^2 \ln f_i}{\partial \theta_r \partial \theta_s} = -\frac{1}{n}\frac{\partial^2 \ln IWL}{\partial \theta_r \partial \theta_s},$$

$$L_{rst} = \frac{1}{n}\sum_{i=1}^{n} \left(\frac{\partial^3 \ln f_i^{w_i}}{\partial \theta_r \partial \theta_s \partial \theta_t}\right)_{\theta = \theta'} = \frac{1}{n}\sum_{i=1}^{n} w_i \left(\frac{\partial^3 \ln f_i^{w_i}}{\partial \theta_r \partial \theta_s \partial \theta_t}\right)_{\theta = \theta'}.$$

By using the laws of large numbers given by Cramér (1947), our conditions are sufficient to conclude that

$$L_r(\boldsymbol{\theta}^0) \underset{P}{\longrightarrow} 0, \tag{38}$$

$$L_{rs}(\boldsymbol{\theta}^0) \underset{P}{\longrightarrow} J_{rs}(\boldsymbol{\theta}^0), \tag{39}$$

and

$$|L_{rst}| < M, \tag{40}$$

as a limiting bound in probability. The proof of the theorem now continues as given by Chanda (1954).

Theorem 2. Let $\hat{\boldsymbol{\theta}}$ denote the vector of IWL estimators and $\boldsymbol{\theta}^0$ the vector of true parameter values. Under Condition I, II, and III, $\sqrt{n}(\hat{\boldsymbol{\theta}} - \boldsymbol{\theta}^0)$ has, asymptotically, a multivariate normal distribution with means zero and variance-covariance matrix $\mathbf{J}_0^{-1}$ as $n \to \infty$, where

$$\mathbf{J}_0 = [J_{rs}]_{k\times k} = \left[\lim_{n\to\infty} \frac{1}{n}\sum_{i=1}^{n} E\left(\frac{\partial \ln f_i^{w_i}}{\partial \theta_r} \cdot \frac{\partial \ln f_i^{w_i}}{\partial \theta_s}\right)\bigg|_{\theta = \theta^0}\right] = -\left[\lim_{n\to\infty} \frac{1}{n}\sum_{i=1}^{n} E\left(\frac{\partial^2 \ln f_i^{w_i}}{\partial \theta_r \partial \theta_s}\right)\bigg|_{\theta = \theta^0}\right],$$

$$\text{i.e. } \sqrt{n}(\hat{\boldsymbol{\theta}} - \boldsymbol{\theta}^0) \sim AN(0, \mathbf{J}_0^{-1}).$$

Especially, when θ is one-dimensional parameter,

$$\sqrt{n}(\hat{\theta} - \theta^0) \sim AN\left(0, 1/\lim_{n\to\infty}\left(\frac{1}{n}\sum_{i=1}^{n} w_i I_i(\theta^0)\right)\right),$$

in which $I_i(\theta^0)$ is the information function of item i.

Proof. Substitute $\hat{\theta}$ for θ in Equation 37, note that $L_r(\hat{\theta}) = 0$, then rewrite Equation (37) as

$$\sum_{s=1}^{k} (\hat{\theta}_s - \theta_s^0) L_{rs}(\boldsymbol{\theta}^0) - \frac{1}{2}\sum_{s,t=1}^{k} (\hat{\theta}_s - \theta_s^0)(\hat{\theta}_t - \theta_t^0) L_{rst} = L_r(\theta^0), (r = 1,\ldots,k),$$

or, in matrix notation,

$$(L + G)(\hat{\boldsymbol{\theta}}' - \boldsymbol{\theta}^{0'}) = \left[\frac{1}{n}\sum_{i=1}^{n} w_i \frac{\partial \ln f_i}{\partial \theta_r}\bigg|_{\boldsymbol{\theta} = \theta^0}\right]',$$

where $\mathrm{L} = [L_{rs}(\boldsymbol{\theta}^0)]$ and $G = \left[-\frac{1}{2}\sum_{t=1}^{k}(\hat{\theta}_t - \theta_t^0) L_{rst}\right]$.L and G are k-square symmetric matrices. So

$$(\hat{\boldsymbol{\theta}}' - \boldsymbol{\theta}^{0'}) = (L + G)^{-1}\left[\frac{1}{n}\sum_{i=1}^{n} w_i \frac{\partial \ln f_i}{\partial \theta_r}\bigg|_{\boldsymbol{\theta} = \boldsymbol{\theta}^0}\right]'. \quad (41)$$

Our conditions are sufficient to obtain conclusions (38) to (40). Thus $L \longrightarrow_P \mathbf{J}_0$ positive definite, $G \underset{P}{\longrightarrow} 0$, since $\hat{\boldsymbol{\theta}} \underset{P}{\longrightarrow} \boldsymbol{\theta}^0$ and $|L_{rst}|$ is bounded. Hence, for large $n, L + G$ may be inverted and $L + G \underset{P}{\longrightarrow} \mathbf{J}_0$, and $(L + G)^{-1} \underset{P}{\longrightarrow} \mathbf{J}_0^{-1}$ by Slutsky's Theorem. From Equation 41

$$\sqrt{n}\left(\hat{\boldsymbol{\theta}}' - \boldsymbol{\theta}^{0'}\right) = (L+G)^{-1}\left[\frac{1}{\sqrt{n}}\sum_{i=1}^{n} w_i \frac{\partial \ln f_i}{\partial \theta_r}\bigg|_{\boldsymbol{\theta}=\theta^{\circ}}\right]', \tag{42}$$

it follows from multivariate form of the Center Limit Theorem (Cramér, 1937) that

$$\left[\frac{1}{\sqrt{n}}\sum_{i=1}^{n} w_i \frac{\partial \ln f_i}{\partial \theta_r}\bigg|_{\theta=\boldsymbol{\theta}^0}\right] \sim AN(0, \mathbf{J}_0).$$

Applying a theorem of Chiang (1956, p.338), we may conclude this demonstration by stating that the asymptotic distribution of $\sqrt{n}\left(\hat{\boldsymbol{\theta}}' - \boldsymbol{\theta}^{0'}\right)$ is multivariate normal with means zero and variance-covariance matrix $\mathbf{J}_0^{-1}\mathbf{J}_0\mathbf{J}_0^{-1} = \mathbf{J}_0^{-1}$, as $n \to \infty$.

The following is about the bias of IWLE, for simplify only one-parameter θ is considered in the probability function.

Theorem 3. BIAS (IWLE (θ)) $= O(n^{-1})$.

Proof. The likelihood function of IWLE is

$$l = l(\theta) = \prod_{i=1}^{m} \left(P_i^{u_i} Q_i^{1-u_i}\right)^{w_i} \cdot \prod_{i=m+1}^{n} \prod_{j=1}^{h} \left(P_{ij}^{u_{ij}}\right)^{w_i}, \tag{43}$$

Let $L = L(\theta) = \frac{\partial \ln l}{\partial \theta} = \sum_{i=1}^{n} \Gamma_{1i}$, where by definition

$$\Gamma_{1i} = \begin{cases} \dfrac{\partial \ln \left(P_i^{u_i} Q_i^{1-u_i}\right)^{w_i}}{\partial \theta}, & 1 \le i \le m \\ \dfrac{\partial \ln \left(\prod_{j=1}^{h} P_{ij}^{u_{ij}}\right)^{w_i}}{\partial \theta}, & m+1 \le i \le n \end{cases}. \tag{44}$$

Then $L(\hat{\theta}) = 0, \hat{\theta}$ is IWLE $(\theta) \cdot L(\hat{\theta})$ can be expanded formally in powers of $\hat{\theta} - \theta$ as follows:

$$L(\hat{\theta}) = \sum_{i=1}^{n} \Gamma_{1i} + (\hat{\theta} - \theta)\sum_{i=1}^{n} \Gamma_{2i} + \frac{1}{2}(\hat{\theta} - \theta)^2 \sum_{i=1}^{n} \Gamma_{3i} + \dots$$

where we define

$$\Gamma_{si} = \begin{cases} \frac{\partial^s \ln\left(P_i^{u_i} Q_i^{1-u_i}\right)^{w_i}}{\partial\theta^s}, & 1 \le i \le m \\ \frac{\partial^s \ln\left(\prod_{j=1}^{h} P_{ij}^{u_{ij}}\right)^{w_i}}{\partial\theta^s}, & m+1 \le i \le n \end{cases},(s = 1,2,\dots).$$

This definition is consistent with (44).

Let $x = \hat{\theta} - \theta$,$\Gamma_s = \sum_{i=1}^{n} \Gamma_{si}$. Let us use the closed form that is always valid:

$$L(\hat{\theta}) = \Gamma_1 + x\Gamma_2 + \frac{1}{2}x^2\Gamma_3 + \frac{1}{6}x^3\Gamma_4 + \frac{\eta}{24}x^4\bar{\Gamma}_5, \quad (45)$$

where $\bar{\Gamma}_5 = \max_{\theta} \Gamma_5$ and $|\eta| < 1$.

Define

$$\gamma_{si} = E\Gamma_{si},$$

$$\varepsilon_{si} = \Gamma_{si} - \gamma_{si},$$

Since $Eu_i = P_i$,$Eu_{ij} = P_{ij}$, we find that

$$\gamma_{1i} = E\Gamma_{1i} = 0, 1 \le i \le n, \quad (46)$$

$$\gamma_{2i} = E\Gamma_{2i} =- w_i I_i = \begin{cases} - w_i \frac{P_i^{'2}}{P_i Q_i}, 1 \le i \le m \\ a_i^2 w_i \left[\left(\sum_{j=1}^{h} jP_{ij} \right)^2 - \sum_{j=1}^{h} j^2 P_{ij} \right], m+1 \le i \le n \end{cases}, \quad (47)$$

$$\gamma_{3i} = E\Gamma_{3i} = \begin{cases} w_i \frac{a_i^2}{(1-c_i)^2} \cdot \frac{P_i^{'}}{P_i^2} (P_i - c_i) \left[2\left(P_i^2 - c_i\right) - P_i (1 - c_i) \right], & 1 \le i \le m \\ a_i^3 w_i \left[3 \sum_{j=1}^{h} jP_{ij} \cdot \sum_{j=1}^{h} j^2 P_{ij} - 2 \left(\sum_{j=1}^{h} jP_{ij} \right)^3 - 2 \sum_{j=1}^{h} j^3 P_{ij} \right], & m+1 \le i \le n \end{cases}, (48)$$

$$\varepsilon_{1j} = \begin{cases} w_i \frac{(u_i - P_i) P_i^{'}}{P_i Q_i}, & 1 \le i \le m \\ a_i w_i \left(\sum_{j=1}^{h} j u_{ij} - \sum_{j=1}^{h} jP_{ij} \right), & m+1 \le i \le n \end{cases}, \quad (49)$$

$$\varepsilon_{2j} = \begin{cases} w_i \frac{a_i c_i}{1 - c_i} \frac{P_i^{'}(u_i - P_i)}{P_i^2}, & 1 \le i \le m \\ 0, & m+1 \le i \le n \end{cases}, \quad (50)$$

Let

$$\gamma_s = \frac{1}{n} \sum_{i=1}^{n} \gamma_{si}, \varepsilon_s = \frac{1}{n} \sum_{i=1}^{n} \varepsilon_{si}.$$

Setting (45) equal to zero, the likelihood equation can now be written in term of the γ_s and ε_s as

$$-\varepsilon_1 = x(\gamma_2+\varepsilon_2) + \frac{1}{2}x^2(\gamma_3+\varepsilon_3) + \frac{1}{6}x^3(\gamma_4+\varepsilon_4) + \frac{\eta}{24}x^4\bar{\Gamma}_5, \quad (51)$$

Since $E\Gamma_{si} = \Gamma_{si}$, $s = 2,3,...$, and $i = m+1,...,n$, it may be seen that each ε_s has the form

$$\varepsilon_s = \begin{cases} \frac{1}{n}\left[\sum_{i=1}^{m} w_i K_{si}(u_i - P_i) + \sum_{i=m+1}^{n} a_i w_i \sum_{j=1}^{h} j\left(u_{ij} - P_{ij}\right)\right], & s = 1 \\ \frac{1}{n}\sum_{i=1}^{m} w_i K_{si}(u_i - P_i) + 0, & s > 1 \end{cases},$$

where K_{si} does not depend on n or on u_i. Since P_i,P_{ij},Q_i and $1-c_i$ are bounded, $0 < w_i < 1$, the K_{si} and thus ε_s is bounded. By assumption (c), the bound of $E\varepsilon_s$ does not depend on n. The same conclusion holds for γ_s.

Since $n^{1/2}x$ is asymptotic normal distribution with zero mean and finite variance from Theorem 2, it follows that $Ex^r (r = 1,...)$ is of order $n^{-r/2}$. A similar statement is true of $n^{1/2}\varepsilon_s$. Thus $E(x^r\varepsilon_s^t) \le \left(Ex^{2r}E\varepsilon_s^{2t}\right)^{1/2}$, so that $E(x^r\varepsilon_s^t)$ is of order $n^{-(r+t)/2}$ $(r,t = 1,2,...)$.

To get the bias of IWLE (θ), let us derive from (51) for the asymptotic standard error of $\hat{\theta}$. Square (51) and take expectations to obtain

$$E\varepsilon_1^2 = \gamma_2^2 Ex^2 + 2\gamma_2 E(x^2\varepsilon_2) + E\left(x^2\varepsilon_2^2\right) + \gamma_2\gamma_3 Ex^3 + \gamma_2 E(\varepsilon_3 x^3) + \ldots \quad (52)$$

We neglect terms $o(n^{-1})$,(52) becomes

$$Ex^2 = E(\hat{\theta}-\theta)^2 = \frac{1}{\gamma_2^2}E\varepsilon_1^2 + o(n^{-1}).$$

By (47) and (49), because of the local independence,

$$E\varepsilon_1^2 = E\left(\frac{1}{n^2}\sum_{i=1}^{n}\varepsilon_{1i}\cdot\sum_{t=1}^{n}\varepsilon_{1t}\right)$$

$$
\begin{aligned}
&= \frac{1}{n^2}E\left(\sum_{i=1}^{m}\varepsilon_{1i}\cdot\sum_{t=1}^{m}\varepsilon_{1t}+\sum_{i=m+1}^{n}\varepsilon_{1i}\cdot\sum_{t=m+1}^{n}\varepsilon_{1t}\right)\\
&= \frac{1}{n^2}E\left[\sum_{i=1}^{m}\frac{w_iP_i^{'}}{P_iQ_i}(u_i-P_i)\cdot\sum_{t=1}^{m}\frac{w_tP_t^{'}}{P_tQ_t}(u_t-P_t)\right]\\
&+\frac{1}{n^2}E\left[\sum_{i=m+1}^{n}a_iw_i\left(\sum_{j=1}^{h}ju_{ij}-\sum_{j=1}^{h}jP_{ij}\right)\cdot\sum_{t=m+1}^{n}a_tw_t\left(\sum_{j=1}^{h}ju_{tj}-\sum_{j=1}^{h}jP_{tj}\right)\right]\\
&= \frac{1}{n^2}\sum_{i=1}^{m}\sum_{t=1}^{m}\frac{w_iw_tP_i^{'}P_t^{'}}{P_iQ_iP_tQ_t}E(u_i-P_i)(u_t-P_t)\\
&+\frac{1}{n^2}\sum_{i=m+1}^{n}\sum_{t=m+1}^{n}a_ia_tw_iw_tE\left(\sum_{j=1}^{h}ju_{ij}-\sum_{j=1}^{h}jP_{ij}\right)\left(\sum_{j=1}^{h}ju_{tj}-\sum_{j=1}^{h}jP_{tj}\right)\\
&= \frac{1}{n^2}\sum_{i=1}^{m}\frac{w_i^2P_i^{'2}}{P_i^2Q_i^2}\mathrm{Var}\,(u_i)+\frac{1}{n^2}\sum_{i=m+1}^{n}a_i^2w_i^2\mathrm{Var}\left(\sum_{j=1}^{h}ju_{ij}\right)\\
&= \frac{1}{n^2}\sum_{i=1}^{m}\frac{w_i^2P_i^{'2}}{P_iQ_i}+\frac{1}{n^2}\sum_{i=m+1}^{n}a_i^2w_i^2\left[\sum_{j=1}^{h}j^2P_{ij}-\left(\sum_{j=1}^{h}jP_{ij}\right)^2\right]\\
&= \frac{1}{n^2}\sum_{i=1}^{n}w_i^2I_i,
\end{aligned}
$$

where

$$I_i=\begin{cases}\frac{P_i^{'2}}{P_iQ_i}, & 1\le i\le m\\ \sum_{j=1}^{h}j^2P_{ij}-\left(\sum_{j=1}^{h}jP_{ij}\right)^2, & m+1\le i\le n\end{cases}.$$

Thus,

$$S.E.(\hat{\theta})^2 = Ex^2 = \frac{\sum_{i=1}^{n}w_i^2I_i}{\left(\sum_{i=1}^{n}w_iI_i\right)^2}+o(n^{-1}). \tag{53}$$

Taking expectation on both sides of (51) yields

$$-E\varepsilon_1 = -\frac{1}{\gamma_2}\left(E\varepsilon_1 + Ex\varepsilon_2 + \frac{1}{2}\gamma_3 Ex^2\right), \tag{54}$$

only the terms of order n^{-1} are to be retained, thus

$$Ex = -\frac{1}{\gamma_2}\left(E\varepsilon_1 + Ex\varepsilon_2 + \frac{1}{2}\gamma_3 Ex^2\right). \tag{55}$$

Also multiply (51) by ε_2 and take expectation to obtain

$$-E(\varepsilon_1\varepsilon_2) = \gamma_2 E(x\varepsilon_2) + E\left(x\varepsilon_2^2\right) \quad ,$$

(56)

in (56) only the terms of order n^{-1} are to be retained. Thus

$$E(x\varepsilon_2) = -\frac{1}{\gamma_2}E(\varepsilon_1\varepsilon_2). \tag{57}$$

Note

$$E\varepsilon_1 = 0, \text{ and } \varepsilon_{2i} = 0, m+1 \le i \le n, \tag{58}$$

From (49) and (50)

$$E(\varepsilon_1\varepsilon_2) = E\left(\frac{1}{n^2}\sum_{i=1}^{n} \varepsilon_{1i} \cdot \sum_{j=1}^{n} \varepsilon_{2j}\right)$$
$$= \frac{1}{n^2} E\left(\sum_{i=1}^{m} \varepsilon_{1i} \cdot \sum_{j=1}^{m} \varepsilon_{2j}\right)$$
$$= \frac{1}{n^2}\sum_{i=1}^{m} \frac{w_i^2 a_i c_i}{1-c_i} \cdot \frac{P_i'^2}{P_i^2}.$$

So

$$E(x\varepsilon_2) = -\frac{1}{\gamma_2} E(\varepsilon_1\varepsilon_2) = \frac{1}{n\sum_{i=1}^{n} w_i I_i} \sum_{i=1}^{m} \frac{w_i^2 a_i c_i}{1-c_i} \frac{P_i'^2}{P_i^2}. \quad (59)$$

Finally, substituting (47),(48),(53),(58), and (59) into (55), we have the bias

$$\text{BIAS}\,(\hat{\theta}) = Ex = \frac{1}{\left(\sum_{i=1}^{n} w_i I_i\right)^2} \sum_{i=1}^{n} \left(\frac{w_i^2 a_i c_i}{1-c_i} \cdot \frac{P_i'^2}{P_i^2} l_i + \frac{1}{2}\gamma_{3i} \cdot \frac{\sum_{i=1}^{n} w_i^2 I_i}{\sum_{i=1}^{n} w_i I_i}\right) = \frac{1}{\left(\sum_{i=1}^{n} w_i I_i\right)^2} \sum_{i=1}^{n} \phi_i, \quad (60)$$

where

$$l_i = \begin{cases} 1, 1 \le i \le m \\ 0, m+1 \le i \le n \end{cases},$$

$$\phi_i = \frac{w_i^2 a_i c_i}{1-c_i} \cdot \frac{P_i^2}{P_i^2} l_i + \frac{1}{2}\gamma_{3i} \cdot \frac{\sum_{i=1}^{n} w_i^2 I_i}{\sum_{i=1}^{n} w_i I_i}.$$

Note that $P_i' = a_i(P_i - c_i)\frac{Q_i}{1-c_i}, \frac{\sum_{i=1}^{n} w_i^2 I_i}{\sum_{i=1}^{n} w_i I_i} < 1$, and γ_{3i} is bounded, so ϕ_i is bounded and the bound of ϕ_i does not depend on n. Since $\sum_{i=1}^{n}\phi_i = O(n)$,$\sum_{i=1}^{n} w_i I_i = O(n)$, BIAS $(\hat{\theta})$ is of order n^{-1}, i.e. BIAS $(\hat{\theta}) = O(n^{-1})$.
